# The Emerging Role of HDACs: Pathology and Therapeutic Targets in Diabetes Mellitus

**DOI:** 10.3390/cells10061340

**Published:** 2021-05-28

**Authors:** Saikat Dewanjee, Jayalakshmi Vallamkondu, Rajkumar Singh Kalra, Pratik Chakraborty, Moumita Gangopadhyay, Ranabir Sahu, Vijaykrishna Medala, Albin John, P. Hemachandra Reddy, Vincenzo De Feo, Ramesh Kandimalla

**Affiliations:** 1 Advanced Pharmacognosy Research Laboratory, Department of Pharmaceutical Technology, Jadavpur University, Kolkata 700032, West Bengal, India; pratik.chakraborty88@yahoo.com; 2 National Institute of Technology, Warangal 506004, Telangana, India; vlakshmij@gmail.com; 3 AIST-INDIA DAILAB, National Institute of Advanced Industrial Science & Technology (AIST), Higashi 1-1-1, Tsukuba 305 8565, Japan; rajkumar-singh@oist.jp; 4 School of Life Science and Biotechnology, ADAMAS University, Barasat, Kolkata 700126, West Bengal, India; gangopadhyaymoumita75@gmail.com; 5 Department of Pharmaceutical Technology, University of North Bengal, Darjeeling 734013, West Bengal, India; ranaju4u@yahoo.co.in; 6 Applied Biology, CSIR-Indian Institute of Technology, Uppal Road, Tarnaka, Hyderabad 500007, Telangana, India; vijaykrishna079@gmail.com; 7 Internal Medicine, Texas Tech University Health Sciences Center, Lubbock, TX 79430, USA; Albin.John@ttuhsc.edu (A.J.); hemachandra.reddy@ttuhsc.edu (P.H.R.); 8 Neuroscience & Pharmacology, Texas Tech University Health Sciences Center, Lubbock, TX 79430, USA; 9 Neurology, Departments of School of Medicine, Texas Tech University Health Sciences Center, Lubbock, TX 79430, USA; 10 Public Health Department of Graduate School of Biomedical Sciences, Texas Tech University Health Sciences Center, Lubbock, TX 79430, USA; 11 Department of Speech, Language and Hearing Sciences, School Health Professions, Texas Tech University Health Sciences Center, Lubbock, TX 79430, USA; 12 Department of Pharmacy, University of Salerno, 84084 Fisciano, Italy; 13 Department of Biochemistry, Kakatiya Medical College, Warangal 506007, Telangana, India

**Keywords:** diabetes mellitus, glucose metabolism, histone deacetylase, HDACs, histone deacetylase inhibitor, HDACi, insulin release, sirtuins, sirtuin activaton

## Abstract

Diabetes mellitus (DM) is one of the principal manifestations of metabolic syndrome and its prevalence with modern lifestyle is increasing incessantly. Chronic hyperglycemia can induce several vascular complications that were referred to be the major cause of morbidity and mortality in DM. Although several therapeutic targets have been identified and accessed clinically, the imminent risk of DM and its prevalence are still ascending. Substantial pieces of evidence revealed that histone deacetylase (HDAC) isoforms can regulate various molecular activities in DM via epigenetic and post-translational regulation of several transcription factors. To date, 18 HDAC isoforms have been identified in mammals that were categorized into four different classes. Classes I, II, and IV are regarded as classical HDACs, which operate through a Zn-based mechanism. In contrast, class III HDACs or Sirtuins depend on nicotinamide adenine dinucleotide (NAD^+^) for their molecular activity. Functionally, most of the HDAC isoforms can regulate β cell fate, insulin release, insulin expression and signaling, and glucose metabolism. Moreover, the roles of HDAC members have been implicated in the regulation of oxidative stress, inflammation, apoptosis, fibrosis, and other pathological events, which substantially contribute to diabetes-related vascular dysfunctions. Therefore, HDACs could serve as the potential therapeutic target in DM towards developing novel intervention strategies. This review sheds light on the emerging role of HDACs/isoforms in diabetic pathophysiology and emphasized the scope of their targeting in DM for constituting novel interventional strategies for metabolic disorders/complications.

## 1. Introduction

Diabetes mellitus (DM), a group of chronic metabolic disorders, is chiefly characterized by a persistently elevated blood glucose level due to deficiency and/or responsiveness of insulin [1]. Among two main types of diabetes mellitus, type 1 DM (T1DM) is caused by the absolute insulin deficiency due to damage of insulin-producing pancreatic β cells, whereas type 2 DM (T2DM) is associated with insulin resistance, which ultimately may give rise to a relative deficiency of insulin [2]. In both cases, interactions between the genes and the environment are responsible for developing the syndrome [3,4]. Additionally, some extracellular inflammatory factors in the islets of Langerhans have been revealed to be involved in diabetic pathogenesis despite their different genetic backgrounds [5]. Insulin deficiency in diabetic subjects can result in divergences of substrate metabolism [1]. This metabolic imbalance is not only restricted to carbohydrate metabolism but can also hamper protein and lipid metabolisms [6]. The T1DM is characterized by the lack of circulating insulin levels but in T2DM, the circulating insulin levels are either normal or slightly elevated or mildly reduced. T2DM accounts for >90% of all diabetic disease and promotes microvascular (retinopathy, nephropathy and neuropathy) and macrovascular complications (cardiovascular comorbidities), due to persisted hyperglycaemia and developing insulin resistance (metabolic) syndrome [7]. It causes huge psychological and physical strains to patients and put an enormous burden on them for healthcare management [8,9]. Both environmental (obesity, unhealthy diet and no physical activity) and genetic factors acquire several pathophysiological perturbations that lead to impaired glucose homeostasis in T2DM; therefore, insulin resistance and impaired insulin secretion are the key defects in T2DM [7,9].

The pathological basis of the metabolic imbalance in diabetic subjects is an absolute or relative insulin deficiency in the blood [10]. Thus, maintaining the healthy mass of pancreatic β cells can be a useful therapeutic aspect in DM [6,10]. Many studies revealed that the restoration of the pancreatic β cell population can restore normal substrate metabolism via triggering insulin signalling and subsequently reduce the chance of developing DM [11,12]. However, the mechanism underlying the alteration of insulin signalling in pancreatic β cells remains unclear.

Recent reports revealed that the role of epigenetic regulation in the development of diabetes [13,14]. However, this field of research is still very young. Histone acetylation and deacetylation are important events for epigenetic gene regulation, which maintain a balance between physiological and pathological states [15,16]. Histone deacetylases (HDACs) comprise a group of enzymes, which catalyze the deacetylation of histone [16]. In the DM, activation of HDACs is rather pathogenic causing disruption of insulin turnover and glucose metabolism in different tissues by multiple mechanisms [6]. HDACs and Histone acetyltransferases (HATs) are involved in chromatin remodeling by catalyzing Histone deacetylation and acetylation, respectively [17]. The combined activities of these two groups of enzymes play a vital role in gene transcription, cell growth, and apoptosis [17]. Cellular signalling and gene expression can be mediated by controlling the HDACs-mediated deacetylation of histone and non-histone proteins [18]. The deacetylation process plays an important role in regulating the metabolism of glucose and maintaining glucose homeostasis both at the cellular and tissue level in active biological systems. For instance, PGC1 (undergoes deacetylation by epigenetic action of SIRT6, eventually promotes the production of glucose in the liver by repressing hepatic gluconeogenesis [19].

## 2. HDACs: A General Overview

Histones are special proteins playing a role in the regulation of transcription by helping DNA to condense into its compact nucleosome form [20]. A histone octamer is made up of eight subunits namely two each of H2A, H2B, H3, and H4 [20]. DNA typically wraps around the histone octamer and the acetylation and methylation of the histone proteins regulate the tightness of this wrapping [20,21]. HDACs are the enzymes responsible for the removal of the acetyl group from histone proteins, thereby promoting tight wrapping that makes the DNA less accessible to the transcription factors [21]. The regulation of histone acetylation and deacetylation is achieved by the antagonistic action of HATs and HDACs [17,22]. HDACs are closely associated with glucose metabolism by regulating insulin dynamics [6]. On the other hand, the mechanism of cell signalling and gene expressions is regulated by the HATs by transferring acetyl groups to the lysine residue on histone and non-histone proteins [17,22]. The basic phenomenon of this acetylation is to neutralize the positive charge at the histone and restrict its local association with the negatively charged DNA [23]. Once the acetyl group is bound to the histone, it serves as a docking site for protein bromodomain [24]. Bromodomains can aid in the remodelling of chromatin, or they can recruit other proteins to do the same [24]. The aforementioned gene function and activity are regulated by histone acetylation and deacetylation, but the net effect of the acetylation status of histone reflects in the regulation of target genes transcription. Thus, histone acetylation can be regarded as an important process for many biological or pathological functions.

### 2.1. The HDAC Family: Classes and Inhibitors

HDACs are the enzymes responsible for the deacetylation of lysine residues at histone proteins and thus regulates their post-translational acetylation [22]. In addition to histone deacetylation, HDACs can also regulate the activities of many non-histone proteins [25,26]. Based on their sequence identity and catalytic activities, 18 HDACs were identified in mammals to date, which have been categorized into four different classes [16] (Table 1). Classes I, II and IV are also known as classical HDACs operate through a mechanism based on Zn^2+^, while class III HDACs or sirtuins (SIRTs) include SIRT1-7, which depend on nicotinamide adenine dinucleotide (NAD+) for their function [27].

Class I HDACs comprise HDAC1, 2, 3 and 8, which are structurally homologous to yeast RPD3 protein [16]. These HDACs can epigenetically regulate cell proliferation, differentiation, and cell cycle progression [28]. Interestingly, the members of HDACs principally contain a nuclear localization signal (NLS) but not the nuclear export signal (NES), so they are localized mainly in the nucleus [17]. However, HDAC3 is an exception in HDAC class I, which also contains NES [16]. The functions of class I HDACs are mediated through the formation of multi-protein complexes. HDAC1-HDAC2 conjugates form a catalytic core of several large complexes, such as co-repressing RE1 silencing transcription factor/neural restrictive silencing factor (CoREST), nucleosome remodelling and deacetylase (NuRD), nuclear receptor co-repressor/silencing mediator for retinoid and thyroid receptor (NCoR/SMRT), and switch-independent 3A (Sin3A) complexes [28]. HDAC3 is found to be conjugated with HDAC4, 5 and 7 and others. In contrast, HDAC8 is the only exception in class I, which can function singly [28]. Class II HDACs comprise HDAC4-7, 9 and 10 and they are homologous to yeast HDA1 protein [28]. Unlike class I HDACs, each member of class II HDACs contains an NLS, an NES, and an extra-regulatory domain [17]. However, depending on the photophosphorylation state, the class II HDACs can move in and away from the nucleus and cytoplasm aided by the presence of both NLS and NES [17]. Class II HDACs can deacetylate both histone and non-histone proteins [28]. Class II HDACs are further subdivided into class IIa (HDAC4, 5, 7 and 9) and class IIb (HDAC6 and 10) based on the organization and orientation in different domains within the proteins [16]. Additionally, Class IIa HDACs can handle only a specific and restricted set of natural substrates [17]. They generally act by recruiting co-repressors or co-activators. Generally, class IIa HDACs exhibit poor deacetylation potential unless associated with class I HDACs. For example, the catalytic activity of HDAC4 depends on its association with HDAC3 within a larger NCoR-SMRT complex and NcoR-SMRT-HDAC3-mediated transcriptional regulation of target genes [29]. In contrast, class IIb HDACs are ultimately involved in non-classical epigenetic roles [30]. Unlike the other classical HDACs, HDAC6 is primarily cytoplasmic in nature, and involves cytoskeleton regulation via deacetylating α-tubulin [29,30]. Class III HDACs comprising SIRT1-7 mimic structural homology with yeast Sir2 protein and also belongs to the family of NAD^+^-dependent deacetylases [31]. SIRTs are responsible for the post-translational modifications of many intracellular proteins either by acetylation or by ribosylation of adenosine diphosphate (ADP) [17]. Nevertheless, SIRTs, facilitate the deacetylation of proteins with the help of energy gained through the hydrolysis of NAD^+^ [17]. SIRT1 can regulate the expression of various genes either directly by recruiting other SIRTs by targeting several transcription factors and co-activator/repressor proteins or indirectly through structural modulation of chromatin [32]. SIRTs were found to have a close association with the development of cancer, metabolic dysregulation, and drug resistance through multiple levels of transcriptional regulation [17,32]. HDAC11, a sole member of class IV, is predominantly located in the perinuclear compartment of lymphoblastoid cells and cytosol of resting CD4^+^ T cells [33]. HDAC11 mainly regulates diverse immune functions, sensitivity in CD4^+^ T cells and inflammation [16,27,33]. Additionally, HDAC11 contains a catalytic domain at the N-terminal region [33]. Similar to the first two classes, class IV HDACs also act by the Zn^2+^-dependent mechanism [27]. Unlike class I and II HDACS, HDAC11 does not interact with NCoR and SMRT; however, it interacts with HDAC6 [33].

Overexpression of HDACs is involved in various forms of cancer and some forms of neurological, inflammatory, and vascular disorders [18,22,27,34]. HDAC inhibitors (HDACis) were intrinsically identified to inhibit cell proliferation in multiple cancer cell lines. These HDACis activities are known to be associated with modulated regulatory tumor suppressor gene activities caused by altered histone acetylation status that is a key event in chromatin remodeling [35]. Underscoring their such potential, multiple HDACis were used as antiproliferative agents following the approval of the FDA, wherein HDACis- vorinostat and romidepsin are two key examples [36]. HDACis are largely considered as epigenetic modulators, the utility of which has been explored in combination with other therapies to synergistically improve the response and toxicity to provide the overall therapeutic benefit to the patient [37].

Based on chemical structures, HDAC inhibitors (HDACis) are classified into five groups: hydroxamic acid derivates, such as trichostatin A, belinostat, practinostat, panabiostat, vorinostat, rocilinostat; short-chain fatty acids, like phenylbutyric acid, butyric acid, valproic acid; cyclic peptides, namely romidepsin; benzamides, such as tubastatin A, tacedinaline, 4SC202, entinostat, mocetinostat; and SIRT inhibitors, like sirtinol, cambinol, EX-527 [28,38] (Table 2). However, most HDACis are found to be effective against multiple isoforms of HDACs by targeting the Zn2+ domain [28]. In contrast, SIRT inhibitors comprise entirely different mechanisms of action [38,39]. Mechanistically, this class of HDACis bind non-covalently to the SIRT active site and block the substrate-binding [39]. Indeed, most of the other HDACis comprise a common “pharmacophore” with a “Zn-binding domain” (for docking in the active site) and a “surface recognition domain” to interact with the residues near the active site and gain entry [40]. Pharmacophore with the domains is joined by a linker or spacer allowing the molecule to lie on the catalytic tunnel of the enzyme [40]. Inhibitors of the classical HDACs, the Zn^2+^-dependent HDACs, have shown promising activity in the intervention of DM and associated complications [41,42,43].

**Figure 1:**
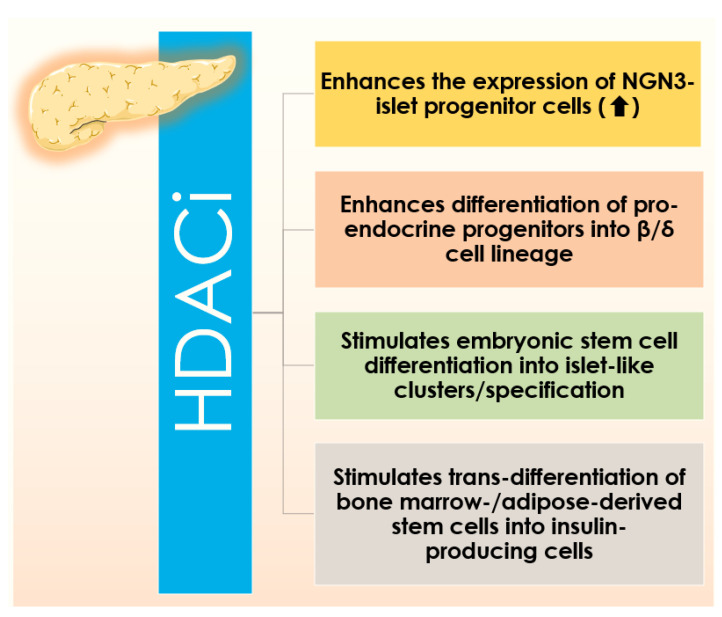
Figure showing different biological activities of HDAC inhibitors (HDACis) that promote the expression of NGN3 and islet progenitor cell numbers, their differentiation into the β/δ cell lineage, embryonic stem cell differentiation into islet-like clusters/specification, and trans-differentiation of bone marrow-/adipose-derived stem cells into insulin-producing cells.

Among the classical HDAC inhibitors, vorinostat, belinostat, romidepsin, panobinostat were approved by the US Food and Drugs Administration (FDA) and Chidamide has been approved by China FDA for therapeutic interventions [28]. Additionally, many of HDACis are under different stages of clinical trials [38]. Most of the aforementioned HDACis are active against multiple isoforms of HDAC and possibly enhancing the chances of developing several untoward effects [28]. In contrast, novel class/isoform-selective HDACis could offer enhanced therapeutic efficacy as compared to non-selective HDACis [28]. Several HDACis have been mentioned to be useful against DM by multiple mechanisms (Figure 1).

### 2.2. HDACs and DM

Both T1DM and T2DM are polygenetic disorders with multifactorial etiologies. Histone acetylation is among the epigenetic processes, which has been revealed to be an important regulatory role in DM [25]. Thus, HDACs can impart significant influence in both types of DM by regulating β cell viability, insulin production, insulin resistance, glucose intolerance, and inflammation [25].

All members of Class I HDACs have been revealed to be associated with insulin resistance [25]. HDAC1 acts as a suppressor of glucose transport through glucose transporter (GLUT) 4 and can interrupt peripheral glucose uptake [25]. The role of HDAC2 in the development of diabetes and associated vascular complications has been well established [44,45,46]. Additionally, it has been pointed out that a significant association between 6q21, the chromosomal location of HDAC2, and DM [45]. HDAC3 has been proposed to trigger hepatic gluconeogenesis [25]. Additionally, single nucleotide polymorphic variants in the HDAC3 gene, such as rs2547547AG and rs2530223CC have been proposed to increase the risk of developing T2DM among the Hans population in China by potentiating HDAC3-mediated aberration in lipid metabolism [47]. In contrast, variant rs11741808AG can lower the risk of type 2 DM by antagonizing HDAC3 [47]. HDAC8 was found to endorse insulin resistance [25]. Additionally, class I HDACs, such as HDAC1 and 3, have been revealed to endorse inflammation in pancreatic β cells [25].

Class II HDACs have been found to regulate the transcription of genes involved in glucose homeostasis and hepatic gluconeogenesis [48]. Activation of class IIa HDACs can endorse the transcription of gluconeogenic genes by deacetylation of forkhead box protein O1 (FOXO1) [48]. HDAC4, 5, 7, and 9 were found to act as metabolic regulators contributing to glucose homeostasis by deacetylation of FOXO1 [49]. Additionally, HDAC4 and HDAC5 can also suppress the GLUT4 gene and contribute to insulin resistance, while HDAC7 has been reported to contribute to the impairment of insulin secretion and trigger β-cell apoptosis [25]. Earlier reports revealed a close association between HDAC9 activation and the development of vascular complications in DM [50,51]. Among class IIb HDACs, HDAC6 has been revealed to be an agonist of glucocorticoids and endorse glucocorticoid-induced hepatic gluconeogenesis [52]. Additionally, HDAC6 can endorse glucose-provoked ROS (reactive oxygen species) generation by regulating acetylation of peroxiredoxin-1 (Pdrx1) and thioredoxin-1 (Trx-1) [53]. HDAC10 deacetylates forkhead box P3 (FOXP3) and thus, can regulate FOXP3-dependent metabolic and inflammatory gene expression [54]. Additionally, HDAC10 in the hypothalamus has been claimed to regulate appetite [25]. Class II HDACs can also associate with GLUT4 at the promoter region and suppress the transcription of the GLUT4 gene in the adipose tissue [55]. It has been shown that GLUT4 transcription can be inhibited due to the loss of histone acetylation of GLUT4 promoter in adult muscle tissues [56]. In contrast, HDAC inhibition can improve GLUT4 transcription resulting in improvement in glucose tolerance and insulin sensitivity [57].

Class III HDACs, SIRTS, are known to regulate energy metabolism and glucose homeostasis [58,59]. The roles of SIRTs in DM remain enigmatic. In most cases, SIRT1, 2, 3 and 6 have been proposed to regulate the genes which trigger insulin responsiveness and glucose homeostasis [59]. Additionally, SIRT1, 2, 3, and 6 have been mentioned to impart beneficial effects by reducing inflammation, suppressing oxidative stress, and restoring mitochondrial functions in the pancreas, skeletal muscle, and adipose tissues [60]. SIRT6 has been revealed to regulate insulin sensitivity and glucose utilization in a positive manner by regulating transient receptor potential vallinoid 1 (TRPV1), calcitonin gene-related peptide (CGRP), and GLUT4 genes [61]. In contrast, SIRT4 and SIRT7 suppress insulin secretion, insulin sensitivity, and fatty acid oxidation by epigenetic regulation. A recent report revealed that increase expression of SIRT5 is associated with the development of DM [62]. However, some contradictory evidence also exists; genetic polymorphisms of SIRT1 and SIRT2 were found to be associated with the development of DM [59]. Polymorphic variants rs10823108 of the SIRT1 gene have been regarded to contribute to the pathogenesis of T2DM [63] and the human SIRT2 gene localized in the 19q13 region of the chromosome is found to contribute as a risk factor in developing T2DM [64]. In a report, SIRT4 expression has been mentioned to reciprocate diabetic nephropathy by reducing oxidative stress and apoptosis [65].

Class IV HDAC, HDAC11, is a relatively new member of the HDAC family, which can preferentially remove fatty acid residues from lysine side chains of a protein or peptide [66]. HDAC11 has been regarded to regulate metabolism and obesity [67]. Deletion of HDAC11 can enhance glucose tolerance, insulin sensitivity, and lipid metabolism [67,68]. Additionally, HDAC11 deletion has been reported to reduce dyslipidemia, oxidative stress, inflammation, and apoptosis in the heart of diabetic mice [69].

Besides T1DM and T2DM, HDACs-suppressed histone acetylation is also related to one form of monogenic autosomal diabetes namely maturity-onset diabetes of the young [45]. Different subtypes of diabetes are associated with the mutation of genes [70]. However, most of these genes encode transcription factors associated with HDACs and HATs and regulate insulin transcription and hepatic gluconeogenesis [70]. Mutations or chromosomal aberration of these genes alter the extent of the interaction of transcription factors with HATs and HDACs [71].

## 3. Role of HDACs in Endocrine Fate of Pancreas

The endocrine cells in the islets of Langerhans of the pancreas consist of mainly α, β, δ, and PP cells producing glucagon, insulin, somatostatin and pancreatic polypeptide hormones, respectively [72]. The cascades of transcription factors and several signalling pathways regulate the growth, development and fate of the endocrine cells of the pancreas [73]. For the proper development of the pancreas, it involves a precise regulation and synchronization of a huge number of transcription factors [73]. Pancreatic and duodenal homeobox 1 (Pdx1) plays a central role in the early development of the pancreas [73]. At an early stage of development, Pdx1-expressing progenitor cells differentiate into endocrine and exocrine cells [73]. Neurogenin 3 (Ngn3) can trigger the initiation of endocrine differentiation from Pdx1-expressing progenitor cells [74]. Ngn3 expressing cells are the islet progenitors [74]. Furthermore, the developments of β and δ cells are linked with the expression of paired box gene 4 (Pax4) [75].

HDACs have been regarded to regulate differentiation of pancreatic progenitor cells and endocrine fates by interfering with the acetylation of histone residues [76]. HDAC1 can silence the expression of β cell-specific Pdx1, which simultaneously obstructs β cell development [77]. HDACis are found to enhances the expression of Ngn3, which consequently enhances the pool of endocrine progenitor cells [78]. The association of HDAC1 with sex-determining region Y box 6 (Sox6) can also prevent β cell proliferation by Sox6 [45]. Among the various HDACis, trichostatin A (TSA) is found to enhance β/δ cell differentiation, while valproic acid is reported to suppress the same [78]. HDAC2 can also interact with Pdx1 at *C*-terminus [79]. HDAC3 can bind to the C/EBP homologous protein (Chop) and activating transcription factor 4 (Atf3) promoters in response to hyperlipidemia associated with T2DM and can induce apoptosis to β cells by endorsing endoplasmic reticulum (ER) stress [80]. On the contrary, Xie and co-authors demonstrated that class I HDACs can promote β and δ cell differentiation from endocrine progenitor cells [76].

Among class IIa HDACs, HDAC4, 5 and 9, can bind with myocyte enhancer factor 2A (MEF2A) to block the transcriptional activity of MEF2A target genes and consequently restrict the development of pancreatic endocrine cells [81,82]. MC1568, a specific class IIa HDACi, treatment to pancreatic explant was reported to increase β and δ cell population by regulating the stability of HDAC–MEF2A complex [82].

Several pieces of evidence revealed the key role of SIRTs in regulating the fates of the endocrine pancreas. Activation of SIRT1 was regarded to promote β-Cell regeneration by triggering endocrine progenitor cells through 5’ AMP-activated protein kinase (AMPK)-mediated activation of Ngn3 and fatty acid oxidation [83]. Additionally, SIRT1 facilitates β-cell formation by endorsing the transcription of Pdx1 mediated through deacetylation of FOXA2 on the promoter of the Pdx1 gene [84]. A recent report revealed that the expression of SIRT3 is required for restoring β cell physiology [85]. SIRT5 has been regarded to regulate the proliferation of pancreatic β cells negatively through suppressing the transcription of Pdx1 by histone H4K16 deacetylation [62,86]. SIRT6 was found to impede the expression of thioredoxin-interacting protein (Txnip) in pancreatic β cells by deacetylating histone H3, thereby it plays an important role in restoring the β cell population [87]. HDAC11 was revealed to function downstream of cytokines in human islets and could endorse the distraction of pancreatic β cells [88]. Thus, it was suggested that HDACs can be the therapeutic target to restore the structural physiology of the pancreas.

### 3.1. Role of HDACs in Regulating β Cell Function and Insulin Secretion

**Figure 2:**
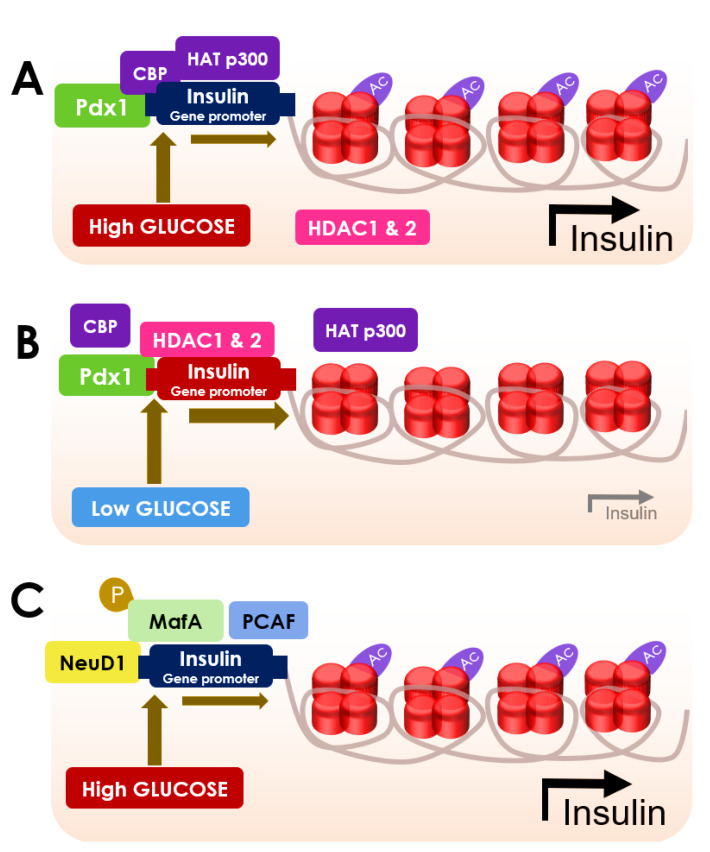
Schematic diagram showing the role of HDACs in regulating β cell function and insulin secretion. (**A**) Acetylation of histone H4 is increased in the insulin promoter when glucose levels are high due to the interaction of Pdx1 with CBP, HAT p300 (acetylated H4, relaxed chromatin and increased insulin expression); (**B**) Pdx1 recruits HDAC 1 and HDAC 2 to the insulin promoter when glucose levels are low, to inhibit H4 acetylation resulting in a decline in insulin production (deacetylated H4, tight chromatin and decreased insulin expression); (**C**) NeuD1 role in promoting insulin gene expression by the acetylation of p300-associated factor (PCAF) and MafA phosphorylation when glucose levels are high.

The function of β cells is to release insulin to maintain glucose homeostasis [89]. An abnormal metabolic milieu can affect β cell development by modifying key regulatory genes, such as Pdx1 and GLUT4 in muscle [90]. Transcription of preproinsulin mRNA from DNA is principally controlled by three specific transcription factors, such as Pdx1, neurogenic differentiation 1 (NeuD1), and V-maf musculoaponeurotic fibrosarcoma oncogene homolog A (MafA) [91,92] (Figure 2). This cycle of transcription and subsequent insulin release from the cells is controlled by glucose itself. The expression of insulin in β cells is regulated by its acetylation [91]. HDACs can regulate the transcription in a negative manner [91].

Pdx1 is known to endorse glucose-provoked insulin gene activation [93]. In hyperglycemic conditions, Pdx1 can recruit p300 and triggers the acetylation of H4 protein [93] (Figure 2). The hyperacetylation of H4 at the proximal promoter region causes the hypertrophy of β cells, which subsequently sets off the transcription of preproinsulin in the insulin promoter region to produce high levels of insulin [45]. High glucose can also trigger the acetylation of H4 at the GLUT2 promoter region [45]. Pdx1 recruits HDAC1 and HDAC2 to the insulin promoter in the hypoglycemic condition, which apprehends H4 acetylation and led to the shutdown of insulin production [93] (Figure 2).

Recruitment of HDAC1 conjugated with the co-repressor Sin3A to the proximal promoter of Pdx1 can cause suppression of Pdx1 expression [45]. HDAC1–Sin3A conjugate can further recruit histone demethylase in a self-propagating epigenetic sequence to suppress histone H3 on lysine 4 trimethylation (H3K4me3), which can suppress Pdx1 transcription [45]. NeuD1 plays a vital role in insulin gene expression through the acetylation of p300-associated factor (PCAF) [94]. Eventually, it serves to promote the binding of the transcription factors to the insulin promoter [94]. Phosphorylation of MafA can facilitate the binding of insulin promoters [94]. Precisely, phosphorylated MafA can interact with PCAF (p300/CBP-associated factor) to increase the transcriptional activity and simultaneously promotes the degradation of MafA [94]. However, the phosphorylation-mediated proteasomal degradation of MafA is delayed in hyperglycemic conditions, which allows an extended period of insulin transcription [91] (Figure 2).

Cyclin-dependent kinase inhibitor 2A (CDKN2A) encoding cell cycle inhibitor genes, such as p16^INK4A^ can epigenetically regulate regeneration and proliferation β cells at the transcriptional and post-translational levels of Cdkn2a locus [95]. HDAC1 can recruit p16^INK4A^ by regulating E2F1 release [96]. HDAC3 is activated through forming stable complexes NCoR1 and SMRT and subsequently suppresses glucose-provoked insulin secretion through regulating several genes [97]. Both pharmacological and genetic inhibition of HDAC3 has been shown to protect β cells and improves insulin gene transcription by triggering suppressors of cytokine signalling 3 (SOCS3) and endorses glucose-provoked insulin secretion [97,98,99].

Class IIa HDACs, namely HDAC4, 5, and 9 possess a distinct ability to hinder the replication of β/δ cell types in the pancreas without affecting α cell mass [81]. Increased expression of HDAC7 has been observed in the pancreatic islets of T2DM patients [99]. HDAC7 has been revealed to inhibit insulin secretion by triggering transcription factor 7-like 2 (Tcf7l2) expression and suppressing the expression of genes regulating DNA replication in β cells [99]. Some reports proposed that the inhibition of HDAC4, 5, 7 and 9 can be fruitful to facilitate insulin production in β cells [45,81,99]. On the contrary, Makinistoglu and Karsenty reported a positive correlation between HDAC4 expression in osteoblasts and circulatory insulin [100]. A recent study by McCann and co-workers revealed that HDAC4 and 5 cannot influence insulin production under normal or hyperglycemic conditions [101]. In another study, mutations in the HDAC4 gene have been revealed to impede β cell functions including insulin production by disrupting the FOXO1 and downregulating β cell-specific transcriptional factors [102]. HDAC inhibition has been reported to increase the expression of insulin 1, insulin 2, GLUT2, and Ogg1 genes [103], while a high dose of HDACis has been implicated to affect negatively in insulin secretory mechanism of the pancreatic β cell line [104].

Class III HDACs, SIRT isoforms, have been described to be the distinct regulators in DM, which can also regulate insulin secretion by pancreatic β cells. SIRT1 can trigger the secretion of insulin by suppressing the uncoupling protein 2 (UCP2) gene and endorsing ATP production in pancreatic β cells [58]. It could also activate the transcription of NeuD, MafA, insulin 1, and insulin 2 to facilitate insulin secretion [58]. Decreased expression of SIRT3 mRNA in pancreatic islets has been correlated to the suppression of Pdx1, MafA, insulin 1, and GLUT2 genes, which consequently results in β cell dysfunction and suppression of insulin secretion [85]. Ectopic SIRT5 expression can inhibit insulin secretion in T2DM by suppressing Pdx1 through H4K16 deacetylation [86]. SIRT6 deficiency has been implicated to impair glucose-induced insulin secretion via deacetylation of FOXO1 and subsequent activation of Pdx1 and GLUT2 expressions [105,106]. SIRT4 gene in pancreatic islets has been proposed to abrogate insulin secretion via interaction with adenine nucleotide translocator 2/3(ANT2/3) and insulin-degrading enzymes under a calorie-sufficient state [58]. SIRT4 has also been revealed to remove three acyl moieties, such as methylglutaryl, hydroxymethylglutaryl, and 3-methylglutaconyl from lysine residues and impede insulin secretion by controlling leucine metabolism [107]. On the other hand, the SIRT7 gene has been anticipated to regulate insulin secretion in a negative manner [59].

To date, substantial evidence is absent regarding the regulatory effect of class IV, HDAC11, on insulin secretion; however, deletion of HDAC11 has been mentioned to suppress high fat-induced hyperinsulinemia in mice [68]. Considering the regulatory effect of HDACs in β cell function and insulin secretion, it could be said that HDACs can be the therapeutic target to alleviate DM.

### 3.2. Role of HDACs in Glucose Homeostasis

**Figure 3:**
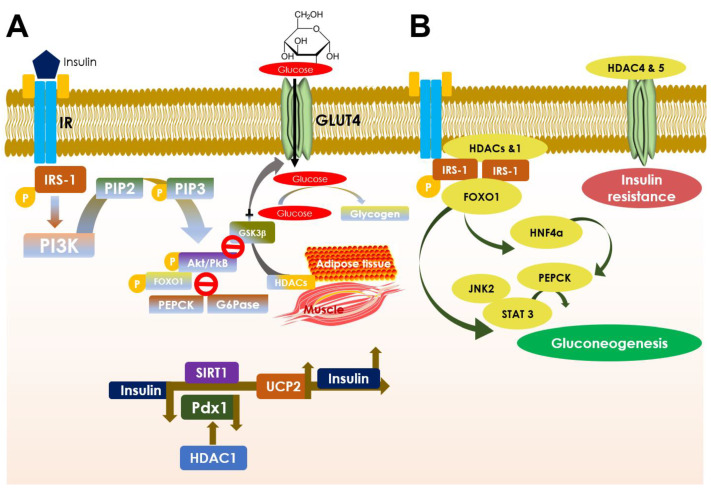
Schematic diagram showing the role of HDACs in glucose homeostasis (**A**) and in developing insulin resistance (**B**). A. Insulin binding at insulin receptor (IR) stimulates IRS-1 substrate phosphorylation and PI3K binding at it that converts PIP2 into PIP3 by phosphorylation under normal physiological condition. PIP3 phosphorylates Akt/PkB and activates its signaling, promoting translocation of GLUT4 to the plasma membrane and activating its function in glucose uptake and glycogenesis. Akt also causes FOXO1-mediated suppression of PEPCK and G6Pase, the two key gluconeogenic genes. Simultaneously, Akt possesses an alternative control on glycogenesis by deactivating GSK-3β and thereby inhibiting glucose to glycogen conversion. HDACs (especially HDAC2 and 5) here control GLUT4 expression and thereby regulate glucose metabolism in adipocytes and muscle cells. The schematic model below shows the role of HDACs, especially controls of HDAC1 on Pdx1 and SIRT1 on UCP2 functions respectively in insulin signaling under differential glycemic conditions. B. HDACs role in developing insulin resistance. HDACs and HDAC1 bind to IRS-1 and limit its phosphorylation and further endorse FOXO1 deacetylation resulting in HNF4α-led induction of PEPCK via JNK2/STAT3 axis, instigating gluconeogenesis in the liver. On the other hand, HDACs, especially HDAC4 and 5, suppress GLUT4 by deacetylation and impede glucose utilization that contributes to developing insulin resistance.

Insulin and glucagon play vital roles in maintaining equilibrium between hepatic glucose production and glucose uptake by the peripheral tissues [108]. Normally, after diet insulin lowers blood glucose by promoting the uptake of glucose by the peripheral tissues and inhibiting hepatic gluconeogenesis [108]. In contrast, glucagon induces endogenous gluconeogenesis and glycogenolysis in the liver [108]. Mechanistically, insulin, after binding to the insulin receptor on the cell surface, promotes autophosphorylation of tyrosine kinases on the β subunit of the receptor and recruits insulin receptor substrate 1 (IRS-1) signal transduction [109] (Figure 3A; Table 1). Eventually, IRS-1 undergoes conformational changes to accommodate phosphatidylinositol 3 kinase (PI3K) by the activated insulin receptor. PI3K is responsible to convert membrane phosphatidylinositol bisphosphate (PIP2) into phosphatidylinositol trisphosphate (PIP3) by phosphorylation [109]. PIP3 phosphorylates protein kinase B (Akt) with the help of PIP3 dependent kinase (PDK) and subsequently triggers the Akt signalling, which further activates GLUT4 by allowing it to translocate to the plasma membrane [109] (Figure 3A). The GLUT4 translocation endorses glucose uptake by glycogenesis [89,109]. On the flipside, activated Akt can also suppress the gluconeogenic genes, such as phosphoenolpyruvate carboxykinase (PEPCK) and glucose-6-phosphatase [110].

The suppression of PEPCK and glucose-6-phosphatase transcriptions is mainly carried out as a result of the FOXO1 [111]. Additionally, activated Akt causes increased uptake of glucose into the cell and converts it into glycogen by glycogenesis [112]. So, Akt simultaneously deactivates glycogen synthase kinase 3β (GSK-3β) for converting glucose to glycogen by glycogen synthase without any interruption [113,114]. Finally, gluconeogenesis and glycogenolysis are downregulated, whereas glycogenesis is upregulated. Activation of Akt can also ensure cell survival via inhibition of pro-apoptotic BAD genes [115]. In contrast, glucagon promotes hepatic gluconeogenesis by upregulating the expressions of PEPCK and glucose-6-phosphatase [116] (Figure 3A).

The regulation of glucose metabolism is largely controlled by post-translational modifications of histones [117]. Acetylation and deacetylation of histone proteins are governed by HATs and HDACs, which are the key players to control gene expressions [117]. HDACs can epigenetically regulate fatty acid and carbohydrate metabolism by regulating the transcription of several genes [17]. Classical HDACs can also influence the deacetylation of non-histone protein, signal transducer, and activator of transcription 3 (STAT3), which results in the suppression of gluconeogenic enzyme expression [17]. Kimura et al. reported that classical HDAC inhibitors can suppress hepatic gluconeogenic enzymes via ER stress inhibition mediated through Janus kinase 2 (JAK2)/STAT3-dependent pathway [118]. Hence, HDACs are the key factors in regulating hepatic gluconeogenesis (Figure 3A,B).

**Figure 4:**
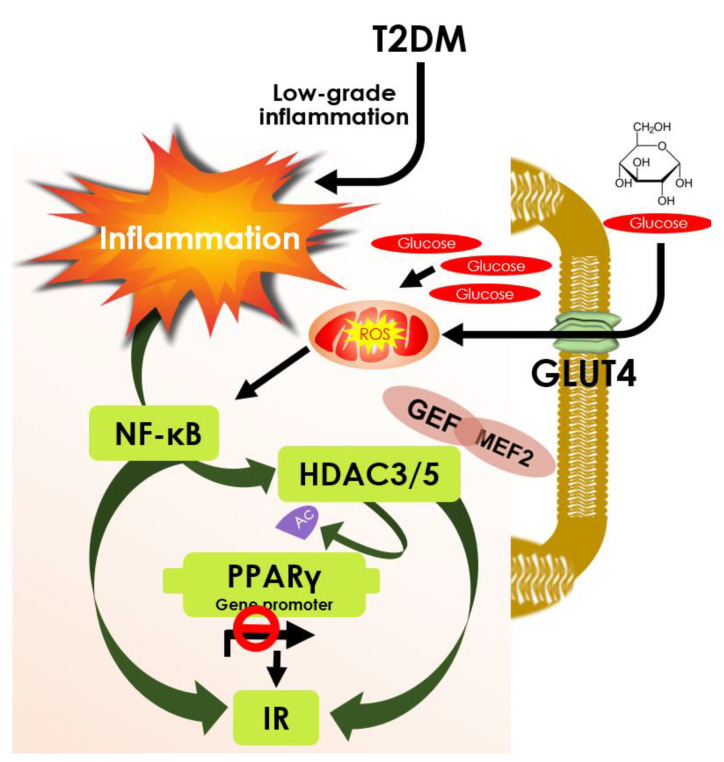
Schematic diagram showing the role of HDAC3 and 5 in PPARγ deacetylation and insulin resistance. Low-grade inflammation in T2DM and Glucose uptake by GLUT4 that produces ROS collectively activates NF-κB function. It modulates HDAC3/5 to deacetylate PPARγ and inactivate its function that contributes to insulin resistance; however, it can be overcome by HDAC3/5 inhibition. GLUT4 Enhancer Factor (GEF) interaction with MEF2A and HDAC3/5 regulates GLUT4 promoter activity acquiring insulin resistance; however, inhibition of HDAC3/5 can increase GLUT 4 expression and prevent insulin resistance.

All members of class I HDACs are regarded as positive regulators in developing insulin resistance [25]. HDAC1 and 2 can suppress insulin signalling by inhibiting the Pdx1 gene [119,120]. Additionally, HDAC1 can suppress GLUT4 and impede glucose utilization. HDAC1, through induction of hepatocyte nuclear factor 4α (HNF4α), induces PEPCK expression and gluconeogenesis in the liver [121]. HDAC2 can also bind to IRS-1 and reduce its phosphorylation as seen in mouse hepatic cells [122]. Activation of HDAC3 epigenetic signature can contribute to developing insulin resistance in T2DM [123]. HDAC3 is known to deacetylate peroxisome proliferator-activated receptor gamma (PPARγ) and subsequently inhibits PPARγ action [124]. On the other hand, HDAC3 inhibition has been shown to reciprocate insulin resistance in adipocytes by activation of PPARγ through its acetylation [124] (Figure 4). Additionally, HDAC3 can endorse FOXO1 deacetylation to facilitate DNA binding of FOXO1 resulting in activation of gluconeogenic genes [125]. Sterol regulatory element-binding protein-1 (SREBP1) can bind to and recruits HDAC8 [126]. Activated HDAC8 can endorse Wnt signalling components resulting development of insulin resistance and glucose accumulation [126] (Table 1).

In a fasting state, class IIa HDACs become dephosphorylated under the influence of glucagon [127,128]. However, on dephosphorylation, class IIa HDACs translocate to the nucleus from the cytoplasm and endorse HDAC3 [17]. HDAC3, in turn, deacetylates FOXO1/3 which facilitates DNA binding of FOXO and increases gluconeogenic gene expressions [17] (Figure 3B). Thus, it becomes evident that class IIa HDACs trigger hepatic gluconeogenesis [125]. HDAC4 and 5 are known to impair GLUT4 expression because of their deacetylation capacity and thus can establish insulin resistance [129]. Deletion of HDAC9 in a hyperglycemic state has been reported to improve glucose tolerance in mice [130]. HDAC6 has been regarded as an essential modifier of glucocorticoid-induced hepatic gluconeogenesis and impair glucose metabolism by regulating nuclear translocation of the glucocorticoid receptor [52]. Thus, inhibition of class II HDACs has been shown to ameliorate insulin resistance and glucose intolerance.

SIRTs, the class III HDACs, are regarded as the key regulators in energy metabolism. In the hyperglycemic condition, SIRT1 can promote insulin-mediated glucose uptake by improving mitochondrial functions and deacetylating of FOXO1 and peroxisome proliferator-activated receptor gamma coactivator 1-alpha (PGC1α) of muscle cells and [59]. Additionally, SIRT1 can improve insulin sensitivity by suppression of uncoupling protein 2 (UCP2) and protein tyrosine phosphatase 1b (PTP1B) [59]. SIRT1 plays a dual role in hepatic glucose production either suppression of hepatic gluconeogenesis by degradation of CREB-regulated transcription co-activator 2 (CRTC2) or activation of hepatic gluconeogenesis by FOXO/PGC1α deacetylation [58]. Unlike SIRT1, SIRT3 can activate PGC1α indirectly, through activating cAMP response element-binding protein (CREB) and AMPK expression [59]. Deletion of SIRT3 has been proposed to develop insulin resistance in mice [62]. In high glucose conditions, SIRT2 can sustain insulin sensitivity and glucose metabolism through augmenting mitochondrial functions and Akt activation [131]. SIRT2 can interact with Akt and subsequently activate Akt signalling in insulin-responsive cells [132]. In another report, activation of SIRT2 has been proposed to improve glucose tolerance and hepatic glucose uptake in the hyperglycemic state via deacetylation K126 of glucokinase regulatory protein in diabetic mice [62]. Additionally, SIRT2 can prevent hepatic gluconeogenesis by inhibiting ubiquitylation of PEPCK through endorsing deacetylation [62]. However, the effect of SIRT2 in insulin signalling is controversial. Arora and dey claimed that SIRT2 can endorse insulin resistance in C2C12 skeletal muscle cells by interfering with the phosphorylation of Akt and GSK-3β [133] Deletion of SIRT5 has been reported to hamper glucose homeostasis by impairing mitochondrial function via hyper-acetylation of metabolic enzymes in brown adipose tissue [134]. SIRT6 deficiency has been revealed to endorse food-induced obesity and insulin resistance [135]. SIRT4 and 7 have been proposed to impede insulin signalling and thus, can contribute to the pathogenesis of type 2 diabetes [25]. SIRT4-mediated dysregulation of AMPK signalling has been implicated to induce insulin resistance by imparting oxidative stress, inflammation and autophagy deactivation [136]. Yu and coworkers reported a positive association between SIRT7 activation and impairment of insulin signalling. SIRT7 can deacetylate FK506-binding protein 51 (FKBP51) and hinder FKBP51-mediated Akt activation by blocking the interaction between pleckstrin homology domain and leucine-rich repeat protein phosphatase (PHLPP) and Akt [137].

Class IV HDAC, HDAC11 is also proposed to regulate metabolic fate by impairing adiponectin-AMPK signalling in the liver [68]. HDAC11 deficiency has been regarded to improve insulin sensitivity by triggering UCP-2 and PGC1α genes [138]. Association between HDAC11 and bromodomain-containing 2 (BRD2) has been implicated to form a regulatory complex to abrogate thermogenic programs in brown adipose tissue. Considering the regulatory effect of HDACs in glucose homeostasis and insulin signalling, it may be suggested that targeting HDACs can be the therapeutic option to alleviate metabolic disorders.

## 4. HDACs: The Possible Therapeutic Targets in DM

Clinical interventions in DM are not only restricted in targeting glucose metabolism but also target many other intermediate processes of substrate metabolism. Apart from the rare exceptions of aberration in the insulin signalling cascade, DM can be ceased by normalizing insulin release from pancreatic β cells [45]. Thus, the preservation of functional β cell mass can be a promising approach to therapeutic interventions of DM. Individual HDACs, including SIRTs, have distinct regulatory roles in pancreatic endocrine development, β cell functions, insulin secretion, and metabolic fates.

Therefore, these are regarded as novel therapeutic targets in DM. Inhibition of classical HDACs has been mentioned to be useful in developing interventional strategies aganist DM [125]. HDACis can inhibit enzymatic activities of classical HDACs (not SIRTs) and thus, influence the transcription of several genes by promoting histone acetylation [139]. Additionally, HDACis can possess secondary effects on other regulatory transcription factors [139]. Inhibition of HDACs has been regarded to regulate the expression of more than 50 histone and non-histone transcription factors. On the other hand, SIRT-boosting therapies have been regarded to be promising interventions against metabolic diseases including DM [140].

### 4.1. Improving β Cell Generation: Via Targeting HDACs

Restoration of the functioning pool of β cells has been regarded to be the principal therapeutic approach in T2DM. Classical HDACis have been found to enhance Pdx1 expressing pancreatic precursors toward Ngn3 expressing pro-endocrine linage and then, HDACis behave in distinct manners based on the inhibition properties [78]. Pan-HDACis (inhibit both classes I and II HDACs), such as sodium butyrate (NaB) and TSA have been revealed to promote the differentiation of pro-endocrine linage into β cells [78]. In contrast, class I selective HDACis, such as valproic acid and MS275, were found to suppress β cell differentiation [78]. In another report, Khan and Jena revealed that valproic acid can improve glucose homeostasis by triggering β cell proliferation, function, and protection [141]. A class IIa HDACi, MC1568, has been found to endorse differentiation and amplification of β cells by enhancing the expression of Pax4 [81]. BRD3308, an isoform-selective inhibitor of HDAC3, has been reported to BRD3308 to endorse β cell proliferation in non-obese female diabetic mice [142].

Harnessing the utility of HDACis has been revealed to have a significant impact in tissue engineering [21]. The inclusion of HDACi, TSA, has been shown to improve the rejuvenating capacity of the pancreatic islet in vitro [103]. Thus, HDACis have been proven to be the key agents in β cell replacement therapy. NaB stimulates early events in embryonic stem cell differentiation to achieve pancreas-like specification, while TSA can facilitate trans-differentiation of bone marrow-derived stem cells to achieve physiological similarity as islet-like clusters [45]. In an ex vivo study, HDAC inhibition by NaB was found to trigger the differentiation of embryonic stem cells into islet-like clusters expressing insulin, glucagon, and somatostatin genes [143]. Recent reports revealed that HDACis can enhance the differentiation of embryonic stem cells into insulin-producing cells [144,145]. In a recent study, valproic acid, a HDACi, was claimed to trigger the differentiation of adipose-derived stem cells into insulin-producing cells which were claimed to be clinically effective against type 1 diabetes [146]. Valproic acid can increase pancreatic endoderm formation [147]. HC toxin, a cyclin peptide HDACi, is reported to promote induced pluripotent stem cell-derived β cells generation by activating the differentiation markers, such as Pdx1, NeuD1, and insulin [147]. The roles of SIRT1, 3 and 6 in restoring β cell mass have been discussed earlier. SRT1720, a SIRT1 agonist, has been shown to endorse the differentiation of endocrine progenitor cells in vitro via triggering AMPK-mediated fatty acid oxidation [83]. Suberanilohydroxamic acid (SAHA; vorinostat), a pan-HDACi, can promote differentiation of stem cells into insulin-secreting cells via activation of SRY-box (SOX17), NK6 homeobox 1 (Nkx6.1), and MafA genes [145].

Activation of SIRT1 by SRT3025 has been reported to cause islet expansion as a consequence of enhancement in β cell mass [148]. γ-Aminobutyric acid (GABA) is known to trigger β cell proliferation via activation of SIRT1 [149]. Resveratrol can promote β-like cell formation from porcine pancreatic stem cells by triggering Wnt/β-catenin signal transduction-mediated through SIRT1 activation [150]. Several naturally occurring small molecules, such as resveratrol, dihydromyricetin, honokiol, 7-hydroxy-3-(4’-methoxyphenyl) coumarin have been reported to activate SIRT3 [134,151,152]. On the other hand, long-chain free fatty acids can trigger the deacetylation capacity of SIRT6 [153]. Thus, these compounds could be tested for their β cell regeneration capacity. SIRT5 is known to negatively regulate β cell proliferation [86]. Kalbas and co-workers developed several peptide-based selective SIRT5 antagonists which could be tested for their effect on β cell proliferation [154].

### 4.2. β. Cell Protection and Promotion of Insulin Secretion: Via Targeting HDACs

Pro-inflammatory mediators, such as interleukin 1β (IL-1β), tumor necrosis factor α (TNFα), and interferon γ (INF-γ) were found to endorse apoptosis of the β cells by triggering several signalling pathways and contribute to the pathogenesis of DM [155]. Pro-inflammatory cytokines have been found to hinder the survival of the transplanted islet grafts in vivo by endorsing inflammation [156]. Pancreatic β cell expresses all 11 classical HDACs, which are differentially regulated by the pro-inflammatory cytokines [88]. Thus, HDACs could be the possible therapeutic targets to prevent structural and functional loss of β cells.

HDACis, such as TSA and SAHA are useful in reciprocating cytokine-induced β cell death and reducing insulin secretion [157]. THS-78-5, a polyaminobenzamide pan-HDACi, has been shown to protect against the IL-1β-mediated loss of β cell viability by attenuating inducible nitric oxide synthases (iNOS) expression and NF-κB (nuclear factor kappa-light-chain-enhancer of activated B cells) trans-activation [158]. ITF2357, a non-specific inhibitor of class I and II HDACs, can protect pancreatic β cells from self-inflicted injury and can promote insulin production by inhibiting endogenous cytokines in the hyperglycemic condition [159]. HC toxin, a class I HDACi, can enhance β cell function and simultaneously promote insulin-triggered glucose uptake via IRS1/PI3K/Akt activation [147,160]. Phenylbutyrate, a pan-HDACi, was found to protect β cells by suppressing ER stress and simultaneously improved β cell function to revert insulin resistance [161]. NaB can alleviate metabolic impairments by preventing structural and functional loss of β cells by suppressing IL-1β/NF-κB/MAPK (mitogen-activated protein kinase) suppression [162]. Inhibitors of class I, IIb and IV HDACs, such as HC toxin, CI-994, PCI24781, LAQ824, PCI34051, and SAHA were found to rescue β cells against cytokine-mediated destruction. On the other hand, isoform-selective inhibitors of HDAC8, PCI34051 and Class IIa-selective HDACis did not offer any protective effect to β cells against cytokine-mediated injury [163]. In contrast, MC1568, a class IIa selective HDACi, has been reported to enhance insulin secretion in islets obtained from humans with T2DM [99]. It can produce this effect by reciprocating β cell dysfunction and apoptosis caused by HDAC7 activation as observed in HDAC7-overexpressed murine β cells [99]. MGCD0103, an isoform-selective HDACi for HDAC1, 2, and 3, and 11, exhibited a protective effect on β cells against oxidative stress, inflammation, and apoptosis by activating SOD (superoxide dismutase) genes mediated through histone acetylation of specificity protein 1 and recruitment of RNA polymerase II [164]. MS-275 and CI-994, selective inhibitors of HDAC1, 2, and 3, have been found to inhibit cytokine-induced apoptosis of murine β cells and enhance glucose-triggered insulin production [165,166]. Plaisance and coworkers revealed that MS-275 can alleviate cytokine-elicited β cell dysfunction and apoptosis caused by a lipotoxic agent, palmitate, by suppressing the ER stress [80]. However, the genetic inhibition approach revealed that only HDAC3 inhibition is sufficient to prevent cytokine-induced β cell damage [80,165]. A highly selective HDAC3 inhibitor, BRD3308, can protect pancreatic β cells against cytokine-provoked apoptosis in vitro and in vivo and promotes insulin release in a hyperglycemic state [142,167]. Additionally, selective HDAC3 inhibitor was claimed to be safe as compared to the inhibitors of HDAC1 and 2 [167].

Class III HDACs, SIRTs, have been regarded to be the key regulators in T2DM by regulating inflammation, oxidative stress, and mitochondrial function [60]. SIRT1, 2, 3, and 6 were revealed to suppress inflammation and oxidative stress [60]. Thus, strategies of activating SIRT1, 2, 3, and 6 can offer attractive therapies in T2DM. SIRT1 deficiency has been reported to cause functional impairment of β cells mediated through the activation of UCP2 [58,168]. In contrast, SIRT1 overexpression can completely prevent cytokine-mediated β cell destruction by impairing the NF-κB signalling pathway [169]. Activation of SIRT1 expression via resveratrol has been found to prevent cytokine-mediated β cell damage and maintained glucose-induced insulin secretion [169]. Another SIRT1 activator, fucoidan, can also prevent oxidative stress-mediated β cell apoptosis and elevates insulin biosynthesis via upregulating glucagon-like peptide-1 receptor (GLP-1R) and Pdx1 [170]. Camel milk-derived lactoferrin has been found to activate SIRT1 along with PPAR-γ and exhibit hypoglycemic and insulin-sensitizing effects in patients with T2DM [171]. 3-(4-methanesulfonylphenoxy)-*N*-[1-(2-methoxy-ethoxymethyl)-1H-pyrazol-3-yl]-5-(3-methyl pyridin-2-yl)-benzamide, a glucokinase activator useful in attenuating hyperglycemia associated with T2DM in mice, can prevent cytokine-provoked β cell apoptosis via SIRT1 activation [172]. Artesunate can prevent cytokine-induced β cell apoptosis via activation of SIRT1 and inhibiting NF-κB, iNOS, NO activities [173].

Nicotinamide mononucleotide, a SIRT1 activator, has been reported to prevent cytokine-induced β cell dysfunction and facilitates insulin secretion. Suppression of SIRT3 in diabetic islet is known to be associated with β cell dysfunction mediated through enhancement of ROS production, IL-1 β synthesis, MAPK activation [174]. SIRT3 activation has been shown to prevent β cell dysfunction and thus, small molecule SIRT3 activators could be the prospective therapeutic agents to treat DM. Earlier reports suggested that SIRT6 activation can improve glucose-induced insulin secretion and thus, pharmacological activation of SIRT6 may serve as the potential therapeutic approach to attenuate DM [62,105]. MDL-800, a selective SIRT6 activator, has been reported to enhance its deacetylation activity to 22-fold by binding to the allosteric site [114]. Cyanidin, a naturally occurring anthocyanidin, can increase SIRT6 activity to almost 55-fold by binding to the β6/α6 loop at the acetyl-lysine binding tunnel [175]. Additionally, long-chain free fatty acids, UBCS039, fluvastatin, methysticin have been reported to activate SIRT6 [153,176,177]. Thus, these compounds may be targeted to find out their potency in improving insulin secretion in response to hyperglycemia. SIRT5 and SIRT7 have been regarded to regulate insulin secretion negatively. Thus, pharmacological inhibition of SIRT5 and SIRT7 could promote insulin secretion. However, the inhibitors of SIRT5 and SIRT7 were also found to inhibit other SIRTs, which necessitate developing isoform-selective inhibitors of SIRT5 and SIRT7 [178]. As discussed earlier, the specific role of HDAC11 in β cell function and insulin secretion has not been revealed; however, HDAC11 activation was found to be associated with elevated cytokine levels in pancreatic islets [88]. Additionally, HDAC11 can result in insulin resistance in high fat-induced hyperinsulinemic mice [68]. Thus, HDAC11 inhibition may have a protective role in DM.

### 4.3. Improving Glucose Homeostasis: Via Targeting HDACs

In recent times, HDACs have emerged as potential new molecular targets for the mitigation and intervention of DM as they play a regulatory role in insulin signalling [42]. HDACs can regulate glucose homeostasis through epigenetic regulation of IRS2 expression and controlling IRS2 activity by regulating H3K9 promoter acetylation [120]. IRS2 can also regulate β cell proliferation [120]. Pan-HDACis, SAHA and TSA can promote H3K9 acetylation in the IRS2 promoter by HDAC1 inhibition [120].

CREB-binding protein and p300 are the critical cofactors in driving β cell genesis, β cell proliferation, and maintaining glucose homeostasis [179]. Additionally, CREB-binding protein and p300 can inhibit hepatic gluconeogenic genes by inhibiting the DNA binding capacity and transcriptional activity of FOXO1 through its acetylation [125]. HDACis, TSA and NaB, can endorse transactivation CREB-binding protein/p300 and thus, may be beneficial in improving glycemic status in DM [180]. Valproic acid can inhibit hyperglycemia by suppressing hepatic gluconeogenic genes, such as glucose-6-phosphatase, fructose-1,6-bis-phosphatase, and phosphoenolpyruvate carboxykinase by inhibiting transcriptional activation of glucocorticoid receptor-mediated through its acetylation [181]. In this study, it has been shown that only class I HDACs, specifically HDAC1 and 3 can interact with glucocorticoid receptor and thus, pan-HDACi and class I selective HDACi were found to be effective in inhibiting glucocorticoid receptor activation, while class IIa selective HDACi possessed only a marginal effect [181]. Class I selective HDACi, such as MS275 can enhance oxidative metabolism in skeletal muscle and adipose tissue by the mitochondrial mechanism mediated through the activation of PGCα and PPARγ/PGC1α signalling in skeletal muscle and adipose tissue, respectively [182]. Chromatin immunoprecipitation assay revealed that the observed effect is associated with HDAC3 inhibition by class I selective HDACi [182].

In contrast, class II selective HDACi, MC1568, was found to be ineffective in regulating energy-dependent oxidative metabolism [182]. Remsberg and co-researchers reported that the selective inhibition HDAC3 has been revealed to improve glucose tolerance by increasing insulin secretion [97]. In another report, inhibition of HDAC3 by butyrate was shown to prevent the development of insulin resistance and obesity in high fat-fed mice via triggering PGC1α/AMPK activation and increasing adaptive thermogenesis and fatty acid oxidation in skeletal muscle and brown fat [183]. HDAC inhibition by TSA, a pan-HDACi, has been regarded to abrogate HDAC2-IRS-1 interaction and trigger tyrosine phosphorylation via IRS-1 acetylation [184]. This observation suggested that specific inhibitors of HDAC2 can improve insulin-dependent glucose transport via IRS-1/Akt activation [184]. HDAC8 can promote insulin resistance [126]. Thus, isoform-selective inhibitors of HDAC8, such as PCI34051, PCI-34058, ITF3056 (givinostat), *N*-hydroxy-3-[1-(phenylthio) methyl-1H-1,2,3-triazol-4-yl]benzamide (NCC149) derivatives etc. [185,186,187] could provide the possible therapeutic options in T2DM by improving insulin sensitivity.

Class IIa HDACs, HDAC4, 5, and 7 are known to activate hepatic gluconeogenesis through dephosphorylation followed by their nuclear translocation, where they can associate with the promoters of gluconeogenic genes [127,188,189]. HDAC4 and 5 can also cause transcriptional induction of these genes via HDAC3 recruitment and HDAC3-mediated activation of FOXO1 and FOXO3 [127]. Thus, inhibition of class I and IIa or pan-HDACis can suppress hepatic gluconeogenesis. HDAC5 can repress the GLUT4 gene and genetic inhibition of HDAC5 has been shown to improve glucose utilization and insulin sensitivity in skeletal muscle cells by endorsing GLUT4 activation [190]. Scriptaid, a pan-HDACi exhibiting IC50 of 2 and 0.6 μM for HDAC5 and Class I HDACs, respectively, has been shown to correct glucose metabolism in skeletal muscle by restoring GLUT4 activity [190]. However, it is not worthy to mention that the effect is directly associated with HDAC5 inhibition [190]. HDAC9 together with HDAC3 can promote hepatic gluconeogenesis provoked by the hepatitis C virus via FOXO1 activation and thus, pharmacological inhibition of HDAC9 has been proposed to be a therapeutic strategy to attenuate hepatitis C virus-induced metabolic abnormality as well as T2DM [49]. Class IIb HDAC, HDAC6, has been revealed to endorse glucocorticoid receptor activation and simultaneously trigger hepatic gluconeogenesis resulting in an impairment of glucose metabolism [52]. In contrast, HDAC6 selective inhibitor, tubacin, can suppress gluconeogenic gene expression in the liver via hyperacetylation of heat shock protein 90 (hsp90) and suppression of glucocorticoid receptor translocation [52]. Thus, selective pharmacological inhibition of HDAC6 can mitigate the diabetogenic effect of glucocorticoids [52]. The comparison between class I and class II HDACis revealed that the class I specific HDACis are superior in regulating energy metabolism and improving insulin sensitivity over class II specific inhibitors, which may be associated with PPARγ activation by class I HDACs [182] (Figure 4).

Class III HDACs or SIRTs are regarded to be the important regulators of glucose and lipid metabolism through interacting with several factors, such as is regarded to regulate PGC1α, SREBP, PPARγ, FOXO1, hypoxia-inducible factor 1α (HIF-1α), CREB-regulated transcription coactivator 2 (CRTC2), AMPK [58]. SRT1720, a small molecule SIRT1 activator, can promote glucose homeostasis (whole-body) and insulin sensitivity in adipose tissue, skeletal muscle and liver as observed in multiple in vivo models of T2DM [191]. Interestingly, SRT1720 was found to inhibit hepatic gluconeogenesis [191]. In contrast, Park and co-workers reported that SRT1720 recruits AMPK to improve glucose homeostasis in a SIRT1 independent manner [192]. In another report, SRT1720-mediated activation of SIRT1 has been proposed to promote insulin sensitivity, glucose tolerance, and lipid metabolism by endorsing angiogenic factors in pre-adipocytes to induce adipose tissue angiogenesis [193]. Vitamin K supplements can reverse hyperglycemia and insulin resistance in patients with T2DM [194]. This effect has been proposed to be mediated through activation of SIRT1/AMPK signalling of glucose metabolism in the liver [194]. SRT501, a less effective SIRT1 activator, can also possess a significant hypoglycemic effect in T2D mice [191]. MHY2233 is a potent SIRT1 activator exhibiting better activation potential than resveratrol and SRT1720 [195]. MHY2233 is a potential pharmaceutical agent in DM, which can improve glucose tolerance by suppressing SREBP1 expression and improves insulin signalling by the IRS-1/Akt pathway [195]. AS101, a tellurium compound, has been found to reverse insulin resistance and attenuate T2DM in rats by triggering SIRT1 activation and deacetylation function [196]. Naltrexone, a TLR4 antagonist, has been found to diminish hyperinsulinemia-mediated insulin resistance via activation of SIRT1 [197].

As discussed earlier, SIRT2 can impart characteristic and even opposing roles in insulin-mediated glucose metabolism. Genetic deletion of SIRT2 has been revealed to reduce insulin responsiveness in the liver and muscle [198]. Nicotinamide mononucleotide (NMN) was found to enhance hepatic glucose uptake by activating SIRT2-mediated deacetylation of K126 of glucokinase regulatory protein in hyperglycemic condition [199]. In contrast, inhibition of SIRT2 by sirtinol can prevent hepatic gluconeogenesis by suppressing PEPCK1 mediated through hyperacetylation [200]. SIRT3 is an important regulator in metabolism and insulin sensitivity and SIRT3 inhibition is regarded to impair insulin sensitivity and augment oxidative stress in skeletal muscle [201]. Celastrol is known to suppress redox stress by endorsing SIRT3 activation along with the recruitment of AMPK/PGC1α signal transduction in the skeletal muscle under diabetic conditions [202]. Honokiol, a SIRT3 activator, has been regarded to reduce oxidative stress and trigger AMPK/CREB/PGC1α activation in Chinese hamster ovarian cells [203]. Thus, it may be effective in reciprocating insulin insensitivity in T2DM. In contrast, inhibition of SIRT3 by berberine has been claimed to reduce hepatic gluconeogenesis by hindering mitochondrial pyruvate import in mitochondria [204]. Similarly, Zhang et al. reported that berberine can promote glucose uptake and inhibit gluconeogenesis by suppressing SIRT3 inhibition [110]. As discussed earlier, several reports claimed that SIRT6 can promote insulin sensitivity and glucose consumption. Anderson et al. revealed a positive association between SIRT6 expression and insulin sensitivity in skeletal muscle and liver in T2DM [205]. SIRT6 activation can also inhibit hepatic gluconeogenesis found to be a potential therapeutic target to develop an interventional strategy against T2DM [19]. Fluvastatin can recruit SREBP-1 and AMPK signalling by direct activation of SIRT6. Thus, it suggests that fluvastatin may serve as a pharmacotherapeutic agent to treat T2DM [177]. Similarly, other SIRT6 activators, such as fucoidan, MDL-800, quercetin, luteolin, pyrrolo-[1,2-a]quinoxaline analogs may be assessed preclinically for the therapeutic potential to correct metabolic abnormalities in T2DM [206]. In contrast, selective SIRT6 inhibitor has also been shown to endorse GLUT1 activation in pancreatic cells and increases glucose uptake by skeletal muscle cells [207]. Sociali and co-workers reported that 2,4-dioxo-*N*-(4-(pyridin-3-yloxy)phenyl)-1,2,3,4-tetrahydroquinazoline-6-sulfonami-de a pharmacological inhibitor of SIRT6, can improve oral glucose tolerance, activate the glycolytic process, and endorse GLUT1 and GLUT4 activation in muscle [208]. Thus, SIRTs serve to be the mystic targets as therapeutic negotiators in regulating glucose homeostasis and insulin sensitivity.

HDAC11 deficiency abrogates obesity and obesity-provoked metabolic syndrome, such as T2DM. Inhibition of HDAC11 is regarded to promote energy expenditure by triggering UCP1 activation in brown adipose tissue [68]. Several small molecules, such as elevenostat, FT895, 2-carboxamidothiophene-based zinc ion chelating carbohydrazides, etc., can selectively inhibit HDAC11 [66]. Additionally, some pan-HDACs, such as romidepsin and TSA have been shown to inhibit HDAC11 in nM concentration [66]. Thus, these compounds could probably be potential agents to correct T2DM by HDAC11 inhibition.

### 4.4. Improving Diabetic Complications: Via Targeting HDACs

#### 4.4.1. HDAC-Mediated Therapeutic Options in Diabetic Nephropathy

Persistent hyperglycemia can affect various tissues and organs resulting in their structural and functional loss [209]. Epigenetic studies revealed that all classes of HDACs play critical roles in diabetic complications by histone modifications [210]. HDAC2, 4, and 5 were found to be upregulated in the diabetic kidney [211]. Thus, HDACs can be potential therapeutic targets in attenuating diabetic nephropathy. Of note, diabetic nephropathy is a major cause of kidney defect/disease and affects ∼40% of T1DM and T2DM patients [212]. Multiple mechanisms in diabetes causing injury to the kidney potentiate its susceptibility among diabetic individuals that leads to nephropathy [213]. Hyperglycemia, hypertension, and genetic predisposition serve as major risk factors in developing diabetic nephropathy, while smoking, hyperlipidemia and amount of dietary protein seem to be the secondary risk factors [212].

**Figure 5:**
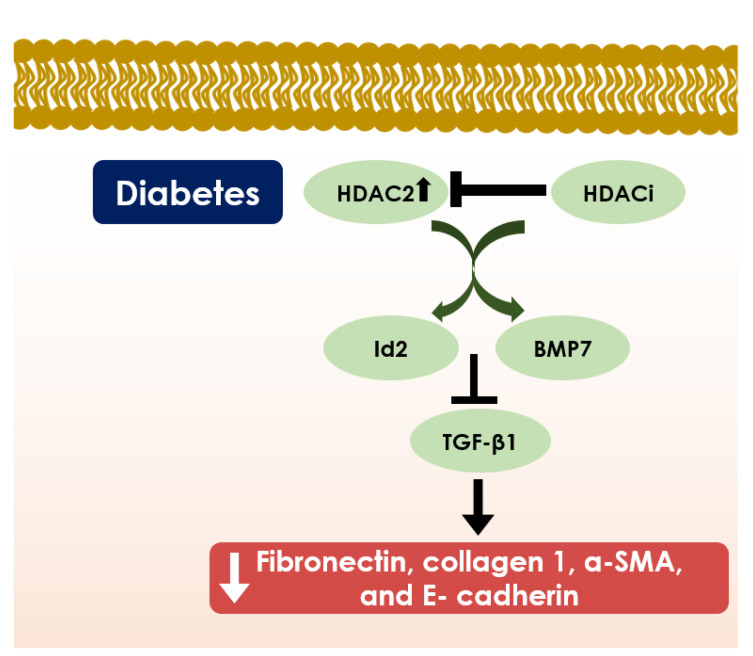
Schematic diagram showing the role of HDACs in diabetic nephropathy. HDAC2 inhibition by HDACi induces expression of DNA binding/differentiation 2 (Id2) and bone-morphogenic protein 7 (BMP7) that represses downstream TGF-β1 signalling and subsequently declines fibronectin, collagen 1, α- SMA, and E- cadherin expressions; and thereby, it provides protection either from diabetic nephropathy or nephromegaly.

HDACs regulate the development and progression of diabetic nephropathy by maintaining acetylation balance on chromatin remodelling and regulating the transcription of genes [211] Long-term oral treatment of SAHA, a pan-HDACi, was found to reduce glomerular hypertrophy, mesangial collagen IV deposition, and albuminuria in diabetic mice by suppressing endothelial nitric oxide synthase (eNOS)-mediated oxidative stress [214]. Additionally, SAHA can reduce diabetes-mediated kidney enlargement by downregulating epidermal growth factor receptor (EGFR) expression [215]. NaB, pan-HDACi, can inhibit diabetic-associated renotoxicity by suppressing hyperglycemia, oxidative stress, fibrosis, inflammation, DNA damage, and apoptosis in the diabetic kidney [162]. NaB was found to suppress eNOS, iNOS, α-smooth muscle actin (α-SMA), collagen I, fibronectin, transforming growth factor β 1 (TGF-β1), NF-κB expression and endorses nuclear factor erythroid 2-related factor 2 (Nrf2), heme oxygenase 1 (HO-1), and NAD(P)H dehydrogenase quinone 1 activation [162,216]. HDACis, such as TSA, valproic acid, and SK-7041 were found to suppress epithelial-to-mesenchymal transition (EMT) in renal tubular cells and inhibit the expression of extracellular matrix (ECM) components in both transcriptional and translational levels in diabetic kidneys [44]. Further study concluded that the effect was mainly associated with HDAC2 inhibition by pan and class I selective HDACis [44]. Among all classical HDACs, NaB was found to suppress only HDAC2 in kidney cells under high glucose conditions and attenuates hyperglycemia-triggered oxidative stress and apoptosis in renal cells [217]. Valproic acid can ameliorate high glucose-induced myofibroblast activation, fibrogenesis and ER stress in the kidneys of diabetic rats by promoting histone acetylation through HDAC inhibition [218,219]. High glucose and advanced glycation end products (AGEs) can endorse HDAC4 activation, which contributes to the prodocyte injury by impairing autophagy and inducing inflammation [211]. HDAC4 silencing has been proven to attenuate diabetic renal injury, which suggests that pharmacological inhibition of HDAC4 with isoform-selective inhibitor could be a therapeutic option in diabetic nephropathy [211] (Figure 5).

Class III HDACs, SIRTs, can also play a crucial role in regulating diabetic nephropathy. SIRT1 has been regarded as a key epigenetic regulator in diabetic kidneys, which regulates the transcription of NF-κB, STAT3, p53, FOXO4, and PGC1α genes [220]. Increased SIRT1 expression either by genetically inducing SIRT1 expression in podocytes or by pharmacological activation through an isoform-selective SIRT1 agonist, BF175, can attenuate albuminuria, oxidative stress in glomeruli, and podocyte injury via multiple downstream signallings [220]. Srivastava and co-workers reported that fibrogenic programming in diabetic kidneys is associated with the abnormal glycolysis caused by SIRT3 downregulation [221]. Additionally, SIRT3 overexpression was found to facilitate ameliorate diabetic nephropathy in engrafted amniotic fluid stem cells of T2D mice by restoring mitochondrial homeostasis and modulating mitophagy [222]. Thus, restoration of SIRT3 expression or pharmacological activation of SIRT3 may mitigate renal fibrosis in diabetic conditions [221]. Pharmacological activation of SIRT3 by liraglutide has been reported to suppress the intrinsic pathway of apoptosis and restore mitochondrial function in renal mesangial cells in diabetic kidneys [223]. Nicotinamide riboside, a SIRT3 agonist, treatment can prevent renal damage in diabetic mice [224]. Aucubin, a naturally occurring glycoside, can reduce oxidative stress and inflammation in renal tissue of T2D rats by endorsing Nrf2 and FOXO3a activation and suppressing NF-κB signalling, which has been proposed to be mediated through the activation of SIRT1 and SIRT3 [225]. SIRT4 activation was found to inhibit high glucose-led free radical production, inflammation, and apoptosis in podocytes and represents a therapeutic option in diabetic nephropathy [65]. Similarly, SIRT6 overexpression is associated with high glucose-induced podocyte injury by inhibiting oxidative stress, inflammation, and apoptosis [226]. Thus, pharmacological activation of SIRT4 and SIRT6 can be regarded as therapeutic strategies in attenuating diabetic nephropathy (Figure 5).

#### 4.4.2. HDAC-Mediated Therapeutic Options in Diabetic Cardiomyopathy

High glucose-induced oxidative stress, AGEs accumulation, inflammation, ER stress, apoptosis and a myriad of pathological signalling events have been implicated to contribute to the development and progression of diabetic cardiomyopathy [227]. All four classes of HDACs are implicated in the epigenetic regulation of associated signallings and gene transcription in diabetic cardiomyopathy [140,228]. Thus, targeting of HDACs and SIRTs can be the novel therapeutic approach against diabetic cardiomyopathy.

NaB, a pan-HDACi, can attenuate diabetic cardiomyopathy, as validated by the reduction in cardiac hypertrophy and inhibition of interstitial fibrosis by enhancing redox defense, suppressing apoptosis, improving angiogenesis, activating GLUT1 and GLUT4 in diabetic myocardium in T1D mice [41]. Additionally, NaB has been reported to attenuate diabetic cardiomyopathy by activating MAPK kinase 3 (MKK3)/P38/p38-regulated protein kinase (PRAK) signalling [229]. HDACs inhibition by TSA can prevent hyperglycemia-provoked myocardial ischemia/reperfusion injury through inhibition of intrinsic apoptosis pathway in diabetic rats via Akt-modulated activation and suppression of FOXO3a and Bim, respectively [230]. Magnesium valproate can alleviate cardiomyopathy associated with T1DM by reducing cardiac hypertrophy, improving hemodynamic functions, restoring estrogen receptor expression and regulating lipoglycemic status [231]. MPT0E014, a pan-HDACi, has been reported to attenuate diabetic cardiomyopathy by modulating cardiac peroxisome proliferator-activated receptors (PPARS), fatty acid metabolism, and proinflammatory cytokines. Additionally, MPT0E014 can improve cardiac function by modulating myocardial autophagy and insulin signalling in T2D rats [232,233]. HDACs inhibition by SAHA is regarded to counteract the oxidative stress and functional changes in cardiomyocytes during the early stage of DM [234]. RGFP966, a specific inhibitor of HDAC3, was found to abrogate the initiation of diabetic cardiomyopathy by endorsing dual-specificity phosphatase 5 (DUSP5) activation by acetylating histone H3 on the DUSP5 primer region leading to an extracellular-signal-regulated kinase (Erk)1/2 activation [150]. Gene therapeutic method to inhibit HDAC4 has been revealed to restrict the progression of diabetic cardiomyopathy [235]. In contrast, Kronlage and co-workers reported that HDAC4 silencing can develop heart failure in T1DM and T2DM, which indicates the protective role of HDAC4 in the diabetic heart [236].

HDAC6 is revealed to be associated with diabetic-induced myocardial ischemia/reperfusion injury mediated through increased oxidative stress by suppressing acetylation of Prdx1, an antioxidant protein [53]. Tubastatin A, an isoform-selective HDAC6 inhibitor, can mitigate hyperglycemia-provoked cardiac dysfunction, cardiac infarction, and free radical generation by facilitating the Prdx1 acetylation [53]. Genetic deletion of HDAC11 was found to attenuate DM-associated cardiac apoptosis, inflammation, and dyslipidemia by attenuating cardiac oxidative stress [69]. Thus, pharmacological inhibition of HDAC11 may be a possible therapeutic strategy in diabetic cardiomyopathy.

SIRTs activator, resveratrol, has been reported to improve diabetes-provoked cardiac dysfunction via activation of SIRT1 and SIRT3 [237,238,239,240,241]. SIRT1 activation by resveratrol can alleviate myocardial injuries in diabetic cardiomyopathy via regulation of mitochondrial function mediated through SIRT1-triggered PGC1α deacetylation and Nrf2 activation [239,240]. Additionally, resveratrol was found to recruit sarcoplasmic Ca^2+^ATPase (SERCA2a) in the diabetic hearts by SIRT1-mediated restoration of SERCA2 promoter activity and improves cardiac functions in diabetic cardiomyopathy [237]. Resveratrol can also attenuate oxidative insult myocardial tissue of diabetic mice by triggering autophagic flux by SIRT1-provoked FOXO1 DNA binding at the Rab7 promoter [238]. Bagul and co-workers reported that resveratrol-mediated SIRT1 activation can reduce oxidative stress alternatively via deacetylation of NF-κB (p65) and histone 3 [242]. Resveratrol can also endorse SIRT3 activation and can prevent collagen deposition in the heart and improved cardiac functions [243]. Additionally, SIRT3 activation has been proposed to inhibit high glucose-provoked mitochondrial injury and cardiomyocyte apoptosis by recruiting FOXO3A/Parkin-mediated activation of mitophagy in vitro [244]. Thus, resveratrol can also prevent cardiac fibrosis via SIRT3 activation. SIRT3 activation by resveratrol has been revealed to preserve mitochondrial oxidative phosphorylation, mitochondrial function, and cellular size in the myocardial tissue of diabetic rats by endorsing deacetylation of a mitochondrial transcription factor by augmenting the mitochondrial DNA binding ability to mitochondrial transcription factor [241]. Phloretin, a dihydrochalcone, has been found to prevent high glucose-induced myocardial damage by inhibiting inflammation and fibrosis in vivo and in vitro by restoring SIRT1 expression in cardiac tissue [245]. l-arginine has been proposed to trigger cardiac SIRT1 expression in diabetic rats and ensured defense against diabetes-mediated fibrosis, apoptosis, and inflammation in myocardial tissue [246]. Apelin, an endogenous peptide ligand of the human G-protein-coupled apelin receptor, gene therapy can correct microvascular insufficiency, cardiac hypertrophy, and heart dysfunction in diabetic cardiomyopathy by endorsing SIRT3 expression [247]. Apelin-mediated SIRT3 activation can also suppress high glucose-provoked oxidative insult and endothelial cell apoptosis in diabetic mice [247]. Polydatin, a naturally occurring small molecule, has been proposed to alleviate high glucose-induced cardiac dysfunction by endorsing autophagy flux and improving mitochondrial bioenergetics through SIRT3 activation in the heart of diabetes mice [248]. Kanwal and colleagues reported that SIRT6 and SIRT3 restore each other’s activity and prevent the development of diabetic cardiomyopathy [106]. SIRT3 has been proposed to maintain SIRT6 expression by reducing oxidative stress; while SIRT6 can restore SIRT3 expression by endorsing Nrf2-dependent transcription of the SIRT3 gene in T2D mice. Among other SIRTs, SIRT5 has been regarded to restore normal metabolism and function of the heart by regulating lysine succinylation [249]; while SIRT2 can regulate microtubule stabilization in diabetic cardiomyopathy in T1D rats [250]. Thus, regulation of SIRT expression may be an alternative therapeutic approach in diabetic cardiomyopathy.

#### 4.4.3. HDAC-Mediated Therapeutic Options in Diabetic Retinopathy

Posttranslational modifications of histones have been regarded to play a key role in developing diabetic retinopathy by regulating the genes affected by chromatin structure [251]. Hyperglycemia can endorse HDAC (HDAC1, 2, 6, and 8) activation in the retina and its capillary cells in diabetic animals, which suppresses histone H3 acetylation [251,252]. Abnormality in H3 acetylation can trigger oxidative stress, apoptosis, and cyto-structural abnormality in retinal tissue [252]. In contrast, Kadiyala and coworkers claimed that acetylation of histones and other proteins in the diabetic retina can trigger pro-inflammatory proteins and contribute to the development of diabetic retinopathy [253]. However, the majority of reports revealed that inhibition of classical HDACs can mitigate diabetic retinopathy. TSA, a pan-HDACi, has been regarded to improve transepithelial resistance in retinal pigment epithelium (RPE) cells in vitro and RPE fluid transport in vivo in hyperglycemic rats. In the same study, specific inhibition of HDAC6 by tubastatin-A exhibited a similar effect in maintaining normal fluid homeostasis in the diabetic retina as observed with TSA, which proposed that HDAC6 inhibition could be a potential therapeutic intervention in diabetic retinopathy [251]. In another report, HDAC6 activation in the diabetic retina, has been regarded to promote oxidative stress through suppression of the Trx-1 [254]. However, HDAC6 inhibition by tubastatin-A was found to alleviate high glucose-provoked oxidative stress in retinal tissue by upregulating Trx-1 [254]. HDAC6 inhibition by TSA or tubacin (an HDAC6 selective inhibitor) can also prevent ischemia and reperfusion-induced retinal neurodegeneration [255]. GLP-1 treatment has been reported to alleviate diabetic retinopathy by inhibiting hyperglycemia-provoked autophagy in the retina of T2D rats mediated through the restoration of GLP-1R expression and HDAC6 inhibition [256]. 5-aza-2’-deoxycytidine and TSA, two pan-HDACis, were found to protect human retinal endothelial cells and retinal pigment epithelial cells from toxic effects of hyperglycemic stimuli in vitro by reciprocating high glucose-induced suppression of pigment epithelium-derived factor and counteracting with inflammatory factors [257].

SIRTs are highly expressed in the retina and were inferred as novel therapeutic targets in diabetic retinopathy [258]. SIRT1 activation has been found to prevent the development of diabetic retinopathy by modulating acetylation of NF-κB (p65), suppression of matrix metalloproteinase-9 (MMP-9), and regulating the transcription of several genes involved in inflammation, vascular growth, oxidative stress, fibrosis, and apoptosis [259,260,261]. Chronic treatment of SRT1720, an isoform-selective SIRT1 activator, has been reported to alleviate diabetic retinopathy by preventing cholesterol accumulation, inflammation, visual dysfunction, vascular degeneration, and neuro-degeneration in the retina of T2D mice [262]. Diabetic rats overexpressing the SIRT1 gene exhibited protection against oxidative stress and apoptosis in retinal tissue, which has been proposed to be mediated through the suppression of hyperglycemia-provoked p66shc activation by SIRT1 [263]. Resveratrol can attenuate high glucose-provoked apoptosis in retinal capillary endothelial cells by activating SIRT1/AMPK/PGC1α signal transduction and is proposed to be an effective therapeutic agent to prevent the pathogenesis in the early stage of diabetic retinopathy [263]. Forskolin can promote SIRT1 activation, which subsequently triggers protein kinase A-mediated suppression of cytoplasmic high mobility group box 1 (HMGB1) in high glucose-exposed human retinal endothelial cells [264]. HMGB1 inhibition can reduce inflammation in the retina vasculature [264]. Fenofibrate was found to attenuate diabetic retinopathy by endorsing SIRT1 activation to reduce the NF-κB activity in the retinal tissues of diabetic rats [265]. Retinal SIRT3 activation has been implicated in the protection of retinal capillary endothelial cells against hyperglycemia-induced damage in vitro and in vivo by endorsing redox defence via deacetylating manganese superoxide dismutase (MnSOD) and suppression of poly (ADP-ribose) polymerase [266]. Genetic deletion of both SIRT3 and SIRT5 in diabetic mice revealed significant evidence in developing inner retinal dysfunction [267]. SIRT6 deficiency in the diabetic retina has been regarded to initiate neurodegenerative processes in diabetic retinopathy by changing glycolytic gene expressions and inducing apoptosis [268]. In contrast, SIRT6 overexpression can ensure neuroprotection in high glucose-exposed Müller glial cells by impeding the acetylation of H3K9 and H3K56 [268]. Thus, pharmacological activation of SIRT3, 5 and 6 may offer therapeutic options in attenuating diabetic retinopathy.

#### 4.4.4. HDAC-Mediated Therapeutic Options in Diabetic Neuropathy

Histone H3 and H4 acetylation is regarded to play important roles in painful diabetic neuropathy [54]. HDAC inhibition is regarded to be a therapeutic option for diabetic peripheral neuropathy. Several preclinical assays claimed that HDACis can be useful in attenuating neuropathic pain by targeting specific epigenetic loci; however, very little literature is available regarding the therapeutic potential of HDACi specifically in diabetic neuropathy. Regenacy Pharmaceuticals, LLC patented (U.S. Patent No. 10,040,769) the use of HDACis for diabetic peripheral neuropathy [269]. Ricolinostat (ACY-1215), an isoform-selective and orally effective HDAC6 inhibitor, was reported to attenuate neuropathic pain and reciprocate metabolic defects in rats with diabetic peripheral neuropathy [269]. Additionally, it can improve mitochondrial transport in the high glucose-exposed neurons in vitro [269]. Inhibition of STAT3 phosphorylation by AG490 results in HDAC1 inhibition which can subsequently upregulate high glucose-mediated defect in Schwann cell autophagy in vitro by triggering the activation of autophagy markers, autophagy-related 3 (Atg3) and microtubule-associated protein 1a/1b-light chain 3 (LC3)-I/II [270].

Several SIRTs, the class III HDACs, activators have been reported to play important therapeutic potential in alleviating diabetic neuropathy. SIRT1 can regulate neuronal cell fate in diabetic neuropathy by regulating metabotropic glutamate receptor (mGluR). Several preclinical studies revealed that SIRT1 overexpression/activation can prevent diabetic neuropathy in experimental animals [116,271,272,273]. SIRT1 has been reported to attenuate neuropathic pain in diabetes through suppression of mGluR1/5 by facilitating H3 acetylation at the promoter region of mGluR1/5-encoding Grm1/5 [271]. On the other hand, mGluR2/3 activation of the SIRT1 axis can protect dorsal root ganglia by preserving mitochondrial oxidative phosphorylation [272,273]. SIRT1 activation by SRT1720 has been found to relieve pain behavior and trigger spinal neuronal activation in rats having painful diabetic neuropathy [271]. Additionally, SRT1720-provoked spinal SIRT1 activation can reduce structural synaptic plasticity of spinal dorsal horn neurons via suppression of growth-associated protein 43 (GAP43), postsynaptic density protein 95 (PSD95), and synaptophysin [116]. Additionally, SRT1720 can reciprocate cognitive dysfunction in T2D rats by suppressing oxidative stress, apoptosis, and inflammation in hippocampal tissue via SIRT1/Nrf2/NF-κB/AMPK-dependent mechanisms [274]. SIRT1 activation by isoliquiritigenin can alleviate oxidative stress and mitochondrial impairment in vitro and in vivo by endorsing PGC1α/FOXO3a/AMPK signalling in experimentally induced diabetic neuropathy [275]. Resveratrol can prevent neurotoxicity by improving redox status and impairing inflammation in diabetic neuropathy by endorsing SIRT1 activation [276]. Additionally, SIRT1 activation by resveratrol has been reported to prevent hyperglycemia-provoked hippocampal neuronal apoptosis via deacetylation of p53 [277]. Li and coworkers reported that berberine can attenuate diabetic encephalopathy by endorsing SIRT1 protein activation and suppressing ER stress in the hippocampus of T2D mice [278]. Additionally, berberine imparts neuroprotective effect by rescuing synapse and nerve-related protein (postsynaptic density protein 95, nerve growth factor, synaptin) expression, suppressing inflammatory factors, reducing hyperglycemia, and improving lipid metabolism in db/db mice [278]. Nicotinamide treatment can reduce the nuclear expression of SIRT2 and slightly activate the nuclear expression of SIRT1 in the brain of diabetic rats and suppressing the development of brain dysfunctions [279]. SIRT2 isoforms 2.1 and 2.2 remain downregulated in the dorsal root ganglia in hyperglycemic conditions, which impair axon regeneration potential [280,281]. Downregulation of SIRT2 or activation of a negative mutant of SIRT2 (SIRT2-H150) can hamper neurite outgrowth. In contrast, SIRT2 activation by nicotinamide adenine dinucleotide can potentiate neurite outgrowth and thus, inhibits diabetic sensory neuropathy [280]. Salvianolic acid A can prevent morphological and functional deficits in peripheral nerves by activation of the SIRT3/PGC1α/AMPK axis in diabetic rats [282]. SIRT3 has also been revealed to prevent axonal apoptosis [283]. Thus, SIRTs may serve as the potential targets to develop therapeutic regime diabetic neuropathy.

#### 4.4.5. HDAC-Mediated Therapeutic Options in Diabetic Endothelial Dysfunctions

Persistent hyperglycemia is implicated in vascular endothelial dysfunctions resulting in lethal complications, such as angiopathy, vascular diseases, stroke, heart disease, chronic kidney failure, venous thrombosis, and others [284]. Inhibition of classical HDACs is regarded to protect human vascular endothelial cells by promoting angiogenesis [285]. HDAC2 can suppress MnSOD expression by binding to MnSOD promoter in hyperglycemic condition and contributes to endothelial dysfunction by endorsing oxidative stress and apoptosis [46]. HDAC2 deletion has been found to prevent high glucose-induced toxic manifestation in human umbilical cord veins endothelial cells in vitro [46]. Thus, pharmacological inhibition of HDAC2 may be a potential therapeutic approach to attenuate diabetes-provoked vascular diseases [46]. High glucose was found to trigger HDAC1 and HDAC2 expressions in aortic smooth muscle cells in vitro and in vivo, which consequently endorse NADPH oxidase (NOX) activation and stimulate ROS formation [286]. The interactions of p300 and HDAC1 and HDAC2 with NOX1, NOX4 and NOX5 promoters have been detected at the active transcription sites in luciferase assays [286]. In contrast, SAHA, a broad spectrum HDACi, has been found to protect aortic smooth muscle cells by preventing ROS formation via inhibition of HDACs [286]. HDAC inhibition by NaB has been reported to mitigate diabetes-provoked oxidative damage in aortic endothelial cells by triggering Nrf2 activation through improving aryl hydrocarbon receptor (AHR) and p300 occupancy at the Nrf2 promoter [287]. SIRT1 activation was found to prevent hyperglycemia-induced endothelial dysfunction via multiple mechanisms. Hyperglycemia causes lysine K81 and histone H3 acetylation of Src homology 2 domain-containing protein (p66Shc), which simultaneously triggers endogenous hydrogen peroxide production and induces redox stress-provoked dysfunction endothelial cells [288,289]. SIRT1 can prevent high glucose-induced endothelial dysfunction by inhibiting oxidative stress in the vascular endothelium mediated through deacetylation of p66Shc [288] [289,290]. 2,6-diisopropylphenol (propofol) has been found to reciprocate hyperglycemia-triggered P66shc activation in human umbilical vein endothelial cells through SIRT1 activation and can prevent endothelial ROS accumulation, inflammation, and apoptosis [291]. SIRT1 activation by resveratrol has been reported to mitigate hyperglycemia-induced oxidative stress and aging in endothelial cells by recruiting mitochondrial antioxidant enzymes by recruiting DNA binding capability of FOXO1 and suppressing acetylation capacity of p300 [292]. Hydroxytyrosol nitric oxide has been found to prevent endothelial dysfunction by decreasing ROS production and improving nitric oxide release mediated through an augmentation in SIRT1 expression in high glucose-exposed human umbilical vein endothelial cells [291]. Trans-δ-viniferin, a phenolic compound, can attenuate endothelial oxidative stress and mitochondrial dysfunction by endorsing SIRT1 activation in high glucose-exposed human umbilical vein endothelial cells [293]. SIRT1 has also been found to inhibit hyperglycemia-provoked apoptosis in endothelial cells in vitro by inhibiting mitochondrial fission factor (Mff)-mediated mitochondrial fission, suppressing c-Jun N-terminal kinase (JNK) activation, and sustaining F-actin homeostasis [294]. SIRT1 prevents endothelial senescence and dysfunction by preventing p53 activation mediated through p53 deacetylation [295,296,297]. SIRT1 activators, resveratrol and SRT2104, can alleviate diabetes-associated endothelial apoptosis by suppressing p53 activation via SIRT1-led p53 deacetylation [296,297]. Resveratrol can also prevent high glucose-triggered apoptosis by reducing oxidative stress and mitochondrial dysfunction in human endothelial cells through SIRT1 activation [298]. Additionally, SIRT1 can attenuate diabetes vasculopathy by suppressing the NF-κB activation through its deacetylation, and thus, inhibits inflammatory disorder [299]. Mesenchymal stem cell-conditioned media can ameliorate diabetes-provoked endothelial dysfunction by improving mitochondrial function by the SIRT1-dependent PI3K/Akt/AMPK/PGC1α pathway [300]. Bone marrow-derived early outgrowth cell-mediated repairing of diabetic endothelial dysfunction requires SIRT1 to improve angiogenesis in vitro and in vivo [301]. Similar to SIRT1, SIRT6 expression is required to maintain endothelial homeostasis [299]. Persistent hyperglycemia can produce oxidative stress to the endothelial cells causing cell senescence, which has been implicated in the pathogenesis of vascular complications in DM. SIRT6 suppression can induce diabetic vascular complications by potentiating oxidative stress-induced endothelial cell senescence [302]. Additionally, SIRT6 inhibition has been proposed to cause DNA damage, cell-cycle arrest, and angiogenic defects [303]. Thus, the SIRT6 activator might serve as a therapeutic option in attenuating diabetes vasculopathy.

#### 4.4.6. HDAC-Mediated Therapeutic Options in Other Diabetic Complications

Diabetic foot syndrome is an end-stage complication in T2DM. Overexpression of HDAC2 has been observed in patients with diabetic foot ulcers, which mainly contributes to endothelial progenitor cell dysfunction and the production of ROS and pro-inflammatory factors in endothelial progenitor cells in hyperglycemic conditions [304]. On the other hand, HDAC2 silencing showed protection against impaired cell proliferation, tube formation, oxidative stress, and inflammation [304]. HDAC2 inhibition can also upregulate SIRT1 expression and ensure SIRT1-mediated protection against diabetic foot ulcers [304]. Resveratrol has been proposed to suppress inflammation, inhibit circulatory disorder, reduce ulceration, improve neuroprotection, and enhance tissue generation capacity in diabetic foot syndrome by SIRT1 activation [305]. The C allele of SIRT1 rs12778366 polymorphism has been regarded as a protective factor in foot complications in patients with T2DM [306]. Poor wound healing contributes to the progression of diabetic foot ulcer disease [307].

Impairment in the wound healing process is a serious concern in diabetes, which often leads to the amputation of limbs in diabetic patients. Inhibition of classical HDACs has been claimed to promote diabetic wound healing [308]. Homeobox protein Hox-A3 (Hoxa3)-treated diabetes-derived macrophages exhibited HDAC inhibition, which quickens the wound healing process by inhibiting inflammatory factors [308]. HDACi, valproic acid, was found to accelerate wound healing in high glucose-exposed porcine corneas and hyperglycemia-suppressed wound closure in T1DM rats by upregulating H3 acetylation [309]. NaB, an HDACi, in combination with epidermal growth factor and platelet-derived growth factor-BB can facilitate the healing of acute cutaneous the wound in diabetic mice [310]. SIRT1, 2, 3 and 6 have been reported to promote wound healing in DM. SIRT1 can promote hyperglycemia-provoked corneal epithelial wound healing involving insulin-like growth factor binding protein-3 (IGFBP3)/insulin-like growth factor-1 (IGF-1)/Akt signalling pathway by p53 deacetylation [311]. SIRT3 loss has been proposed to delay the wound healing process in the insulin-resistant mice model of T2DM [312]. SIRT6 depletion in cutaneous wounds has been reported to aggravate the wound healing process by promoting NF-κB activation, inducing oxidative stress and decreasing angiogenesis in diabetic mice [313]. MC2562, a SIRT1, 2 and 3 activator, was shown to accelerate wound repair [314]. SIRT1 activation by resveratrol endorsed its endothelial protection and pro-angiogenic effects, facilitating diabetic wound healing by inhibition of FOXO1 and endorsing c-Myc expression [315]. Inclusion of SRT1720, a SIRT1 agonist, in an embryonic artery CD133^+^ cell-seeded dressing material composed of poly (lactic-co-glycolic acid), collagen and silk has been found to accelerate the wound healing process and induces angiogenesis in diabetic ischemic ulcers [316]. SRT1720 inclusion can potentiate embryonic artery CD133^+^ cell proliferation, enhancing the secretion of vascular endothelial growth factor (VEGF) A, basic fibroblast growth factor, IL-8 and, and inhibiting the TNF-α secretion [316]. Localized use of SRT1720 can promote wound healing and angiogenesis in T1D mice. The effect has been proposed to be associated with the suppression of high glucose-induced oxidative stress as observed in in vitro assay performed in human umbilical vein endothelial cells [317]. Micro-RNA-92a inhibition was reported to promote skin repair in diabetic wound healing by SIRT1 activation [318].

HDACs are known to regulate several pathological events in cardiometabolic diseases including atherosclerosis by inducing inflammation and oxidative stress [319]. Significant upregulation of HDACs was reported in atherosclerotic arterial specimens in diabetic and hypercholesterolemic mice [286,319]. Additionally, HDAC9 can endorse atherogenic plaque formation by suppressing cholesterol efflux and generating activated macrophages in atherosclerosis [320]. Additionally, HDAC9 can enhance the progression of vascular diabetic calcification that can contribute to diabetic atherosclerosis [50]. Inhibition of classical HDACs has been regarded as a therapeutic option in treating atherosclerosis [319]. SAHA, a pan-HDACi, has been found to alleviate atherosclerotic lesions by impeding NOX expressions, reducing oxidative stress and suppressing inflammation in diabetic and apolipoprotein E-deficient hypercholesterolemic mice [286,319]. Inhibition of HDAC2 and HDAC3 by valproic acid can prevent cholesterol absorption by inhibiting NPC1L1 expression, which has been implicated in the development of atherosclerosis associated with DM [321]. RGFP-966, an isoform-selective HDAC3 inhibitor, can significantly reduce diabetic liver damage and aortic pathologies in T1D mice by restoring the synthesis of hepatic fibroblast growth factor 21 through hepatic Nrf2 activation [121]. Among all SIRTs, SIRT1 and SIRT6 are characterized for their protective mechanism against inflammation, vascular aging, and atherosclerosis in DM [322]. SIRT1 expression in peripheral blood mononuclear cells is negatively correlated with pro-inflammatory cytokines level in T2DM patients with coronary artery diseases [323]. Thus, there may be a regulatory role of SIRT1 in developing atherosclerotic lesions [323]. SS-31, a mitochondrial antioxidant, can enhance SIRT1 level and ameliorates leukocyte-endothelium interactions, inflammation, and oxidative stress in T2DM to prevent the risk of developing diabetic cardiovascular diseases, including atherosclerosis [324]. Vascular calcification has been implicated in the risk of cardiovascular disease in DM, which accelerates atherosclerotic plaque development [325]. SIRT1 can negatively regulate vascular calcification in diabetes [325]. SIRT1 activation by SRT1720 can suppress osteogenic differentiation of human vascular smooth muscle cells by inhibiting RUNX2 signalling and preventing senescence [325]. SIRT6 has been revealed to be a negative regulator in endothelial dysfunction and atherosclerosis development [326]. SIRT6-knocked down and apolipoprotein E-deficient hypercholesterolemic mice tends to develop atherosclerosis by increasing inflammation in the endothelial cells [326]. Incretin treatment attenuates diabetic atherosclerosis in T2DM patients; however, SIRT6-attenuated inflammation in atherosclerotic lesions was regarded to potentiate the therapeutic effect of incretin [327].

Cerebral ischemia yields severe morbidity and high mortality in diabetic patients. Classical HDACs are implicated in the neurological disorder, including ischemic stroke by inducing oxidative stress, ER stress, apoptosis, inflammation, and blood–brain barrier breakdown, which have been reported to be reciprocated by classical HDACis, such as TSA, valproic acid, butyrates, and SAHA [328]. HDAC3 has been regarded to be a positive regulator in developing diabetic stroke, which remains upregulated in the brain of diabetic subjects [329]. Inhibition of HDAC3 by a specific inhibitor, RGFP966, can prevent hyperglycemia-induced cerebral ischemia-reperfusion injury in vivo and in vitro by suppressing oxidative stress, inhibiting apoptosis, and improving autophagy [329]. Tubacin, a selective HDAC6 inhibitor, was found to alleviate ischemic brain injury in T2D mice with focal cerebral ischemia by endorsing eNOS expression in an HDAC6-dependent mechanism [330]. Class III HDACs are also considered as the key regulators in diabetes-triggered ischemic brain injury. SIRT1 imparts a neuroprotective role in the brain against cerebral ischemia and neurodegeneration [223,331]. Dexmedetomidine attenuates diabetes-exacerbated cerebral ischemia/reperfusion injury in vitro and in vivo [332]. Dexmedetomidine can alleviate oxidative stress, inflammation and apoptosis in high glucose-exposed mouse hippocampal neurons and middle cerebral artery region in diabetic mice by endorsing SIRT1, Nrf2, and nuclear factor of activated T-cells 5 (NFAT5) expression [332]. *N*-palmitoylethanolamide-oxazoline attenuates ischemia/reperfusion brain injury and cognitive impairment in diabetic rats by SIRT1 activation resulting in attenuation of cerebral inflammation and oxidative stress [333]. Hyperglycemia can suppress SIRT3 expression in the cerebral tissue, which augments neural oxidative stress, inflammation and apoptosis [334]. Honokiol, a pharmacological activator of SIRT3, was shown to abolish neural dysfunctions after intracerebral hemorrhage in T1D rats by decreasing PYD domains-containing protein 3 inflammasome activation, suppressing IL-1β level, and potentiating SOD2 deacetylation and ROS scavenging [334].

## 5. Challenges and Future Prospects

HDACs have emerged as potential targets for the treatment of DM. HDACs-mediated epigenetic and post-translational regulation of several transcription factors that modulate multiple signalling events in DM and the pathological development of diabetic complications serve as the potential mechanism(s) to intervene. Substantial evidence revealed that HDACs can regulate β cell fate, insulin release, insulin expression, insulin signalling, and glucose metabolism. HDACs have also been implicated in the regulation of oxidative stress, inflammation, apoptosis, fibrosis, and other pathological events that essentially contributes to the diabetes-provoked pathogenesis in several tissues. The enhanced expression of the majority of classical HDAC isoforms has been regarded to play pathogenic roles in developing DM and DM-associated complications. In contrast, most of the class III HDACs, such as SIRT1, 2, 3, and 6 were claimed to impede insulin resistance and impart beneficial effects by suppressing inflammation, oxidative stress, and mitochondrial dysfunctions in the DM-affected tissues. Simultaneously, several controversial claims are also reported. However, it is obvious that the targeting of HDACs can provide a theoretical basis to intervene in DM and its associated complications. Emerging evidence revealed the preclinical success of several classical HDACis and SIRT activators in the experimentally induced T1DM, T2DM and DM-led vascular complications in vitro and in vivo. However, the majority of the studies employed non-selective inhibitors and activators of HDACs and SIRTs respectively. Despite some preclinical studies included genetically mutant cells or animals to predict the exact HDAC isoform for the observed effect, but these are too insignificant to portrait the specific roles of the individual members in all four classes of HDACs. Thus, the majority of the evaluations remain confined within the preclinical level.

Metabolic aberration and consequent pathological events in DM are generally manifested by multiple molecular mechanisms involving several transcription factors. All 18 HDACs are implicated in the removal of the acetyl group from ε-lysine residue of proteins and thereby, HDACs can not only impact transcriptional mechanics but can also concurrently endorse posttranslational modifications of proteins. Substantial evidence revealed that modifications in histone acetylation status can regulate the activity of several transcription factors that play a vital part in the pathological or preventive events in DM. Expression of individual HDAC in diabetic patients followed by preclinical assays predicted the regulatory mechanism(s) of different HDAC isoforms in the DM. However, the precise role of individual HDACs is yet to be categorically understood. Additionally, the complex regulatory network of HDACs, all together, must be precisely elucidated. Once the complete understanding of HDACs regulation is characterized, it can potentially serve as a novel therapeutic target for DM and associated pathological complications.

## 6. Conclusions

This review provides insights into the emerging roles of all four classes of HDACs in the developmental and pathogenetic pathways associated with DM. This report provides an up-to-date account of details on the involvement of HDACs in DM etiology and pathology. The molecular insights behind the HDAC-mediated epigenetic regulation in endocrine islet fate, insulin expression, insulin signalling, and glucose homeostasis are comprehensively accentuated. It also discussed the targeting of HDACs for the potential treatment of DM and diabetes-associated complications, such as nephropathy, cardiomyopathy, retinopathy, neuropathy, vasculopathy, foot syndrome, poor wound healing, and cerebral strokes. Largely, the inhibition of classical HDACs and the activation of class III HDACs or SIRTs were described and its therapeutic potential for the DM and associated complications were further extensively discussed, while several controversial pieces of evidence were also conferred. Although this evidence so far distinctly underlined the crucial part HDACs play in DM and associated complications, the complete functional map of HDACs is yet to be revealed. Conclusively, further extensive efforts are warranted to reveal the distinct role of individual HDAC family members in disease pathology towards developing effective HDACs-targeted therapeutic interventions for DM.

## Figures and Tables

**Table 1:** Localization, expression, functions of different HDACs. Their specific roles in respective classes and in glucose metabolism and diabetes are also described.

Classes	Members	Localizations	Expressions	Functions	Specific Function in Glucose Metabolism and Diabetes
Class I (Zn^2+-^dependent)	HDAC1, 2, 3, 8	Nucleus of all types of tissues.	Ubiquitous	Promotes proliferation, IFN signalling, and HIF1α function. Contrary, it represses MTA1 & NF-κB functions.	HDAC1: Acts as repressor for the transcription of GLUT4 HDAC2: Binds to IRS-1 leading to reduced acetylation and decreased Insulin receptor mediated tyrosine phosphorylation of IRS-1 HDAC3: Stimulates Glucose production by FOXO transcription factor. HDAC8: Upregulated in obese mice models and promotes Insulin resistance.
Class II (Zn^2+-^dependent)	HDAC4, 5, 6, 7, 9, 10	Nucleus and cytoplasm of brain, heart, skeletal muscle, pancreas, liver, kidney, and placenta.	Specific	Promotes HIF1α function and HSP 90 function (chaperone). It represses II5 promoter and inhibits Foxp3 and Treg function	HDAC4 & HDAC5: Repress transcription of GLUT4, involved in recruitment of class I HDAC3 to liver and stimulation of glucose production. HDAC6: Stimulates glucocorticoid-stimulated glucose production and contributes to hyperglycemia and glucose intolerance. HDAC7: Levels of HDAC7 are elevated in islets of T2D patients, elevated levels of HDAC7 lead to impairment in Insulin secretion and increased β-cell apoptosis in vitro HDAC9: Suppression of HDAC9 expression leads to improvement in Insulin sensitivity and glucose tolerance
Class III / SIRTUINS (NAD^+^-dependent)	SIRT1-7	Mitochondria, nucleus, and cytoplasm. Brain, and in all the oncogenic tissues.	Ubiquitous	Promotes immune function across various types synapses by NAD^+^-dependent mechanism through SIRT1.	SIRT2 and SIRT4: SIRT2 impair insulin signaling and may contribute to pathogenesis of T2D
Class IV (Zn^2+-^dependent)	HDAC11	Cytoplasm and nucleus of heart, brain, kidney, and skeletal muscle.	Ubiquitous	Inhibits II5 promoter activity in APCs.	NA

**Table 2:** Table showing different HDACi categorized based on their class, chemical identity, specificity, and HDAC class selectivity.

Specificity	Classification	Candidate HDACi Compounds/Drugs	HDAC Class Selectivity
**Pan-HDACis**	Hydroxamates	Vorinostat (SAHA)	class I, II and IV
		Belinostat(PXD-101)	class I and II
		Panobinostat (LBH-589)	class I, II and IV
		Trichostatin A (TSA)	class I and II
		Quisinostat (JNJ-16241199)	class I and II
		WW437	HDAC 2 and 4
	Benzamides	Entinostat (MS-275)	class I
		Tacedinaline (CI-994)	class I
		Mocetinostat (MG-0103)	class I and IV
	Aliphatic fatty acids	Pivaloyloxmethyl butyrate (AN-9)	class I and IIa
		Sodium Butyrate (NaB)	class I and IIa
		Sodium Phenylbutyrate (4-PB)	class I and IIa
		Valproate (valproic acid)	class I and IIa
	Electrophilic ketones	trapoxins(TPX)	class I
		a-ketoamides	NA
		heterocyclic ketones	NA
	SIRT inhibitors	cambinol	SIRT1 and 2
		EX-527	SIRT1 and 2
		sirtinol	SIRT1 and 2
		nicotinamide	class III
	Cyclic inhibitors/peptides	Romidepsin (depsipeptide, FK228)	class I
	Other compounds	Diallyl Trisulfide (DATS)	NA
**Class-specific HDACis**	Benzamides	Ricolinosta(ACY-1215)	HDAC 6
		tubacin	HDAC 6
	Hydroxamate derivatives	Azelaic Bishydroxamic Acid (ABHA)	HDAC 3
		CBHA (m-carboxycinnamic acid bis-hydroxamide)	HDAC 3
	SIRTs inhibitors	SEN196	SIRT1
		COMPOUND 6J	SIRT2
		JGB1741	SIRT1
	N/A	I-7ab	HDAC 3
		RGFP966	HDAC 3
		PCI34051	HDAC 8
		C149	HDAC 8
	Polyketides	Depudecin	HDAC 1
**Bromodomain HDACis**	BET inhibitors	JQ1	BRD4
		I-BET	BET
		BY27	BD2
Hybrid HDACis	Chimerics	CUDC907	HDAC /PI3K
		CUDC101	EGFR/Her-2/HDAC 1

## Data Availability

Not applicable.
